# A novel, FFT-based one-dimensional blood flow solution method for arterial network

**DOI:** 10.1007/s10237-019-01146-0

**Published:** 2019-04-06

**Authors:** Igor Sazonov, Perumal Nithiarasu

**Affiliations:** Biomedical Engineering Group, Zienkiweicz Centre for Computational Engineering, College of Engineering, Swansea University, Bay Campus, Fabian Road, Swansea, SA2 8PP UK

**Keywords:** Fast Fourier transform (FFT), Perturbation method, 1D arterial haemodynamics, Pulse wave propagation

## Abstract

In the present work, we propose an FFT-based method for solving blood flow equations in an arterial network with variable properties and geometrical changes. An essential advantage of this approach is in correctly accounting for the vessel skin friction through the use of Womersley solution. To incorporate nonlinear effects, a novel approximation method is proposed to enable calculation of nonlinear corrections. Unlike similar methods available in the literature, the set of algebraic equations required for every harmonic is constructed automatically. The result is a generalized, robust and fast method to accurately capture the increasing pulse wave velocity downstream as well as steepening of the pulse front. The proposed method is shown to be appropriate for incorporating correct convection and diffusion coefficients. We show that the proposed method is fast and accurate and it can be an effective tool for 1D modelling of blood flow in human arterial networks.

## Introduction

Mathematical and numerical modelling of blood flow in a human arterial network allows researchers to understand various flow related phenomena and disorders. It can help us to understand the genesis and progression of various diseases in the cardiovascular system, and it also provides us with a platform for developing methods for detecting such diseases. The arterial haemodynamics is influenced essentially by pulse wave propagation phenomena. At present time, one of the most effective and fruitful ways to understand wave phenomena in an arterial network is through one-dimensional (1D) flow modelling. Recent works on 1D blood circulation modelling have demonstrated its accuracy in predicting various flow quantities (Mynard and Nithiarasu 2008; Swillens et al. 2008; Alastruey et al. 2011; Gamilov et al. 2014; Sazonov et al. 2017).

The core of the 1D modelling is based upon numerical solution of nonlinear partial differential equations (PDE) derived, for example, in Formaggia et al. (2001), Sherwin et al. (2003). There are many numerical schemes/time integration methods available, including Finite Difference (FD) (Olufsen et al. 2000; Smith et al. 2002; Reymond et al. 2009; Saito et al. 2011), Finite Element (FE) (Formaggia et al. 2003; Sherwin et al. 2003; Mynard and Nithiarasu 2008; Alastruey et al. 2011), Finite Volume (FV) (Cavallini et al. 2008; Delestre and Lagrée 2013) and Discontinuous Galerkin (DG) methods (Matthys et al. 2007; Marchandise et al. 2009; Alastruey et al. 2011). Some of them are compared in the benchmark paper by Boileau et al. (2015). A rather fast scheme is proposed in Carson and Van Loon (2017) which is based on an Enhanced Trapezoidal rule method (Kroon et al. 2012). Many of these works appear to use slightly different mathematical models and constitutive relations as shown later in the present work.

Majority of the existing models, however, assume the convection velocity to be average over the cross section of an artery. Although this approximation is generally accepted, such approximations appear to give incorrect results, even for a steady-state flow. This error is pronounced in highly time-dependent flows, such as the pulsating flow experienced by an arterial network. In addition, space–time methods do not account correctly for the vessel skin friction of an arbitrary pulsating wave in an artery. The viscous effects should be based on the use of the Womersley solution (Womersley 1955) that is too complicated for the 1D space–time approach to handle. Other unsolved issues of 1D space–time approach include difficulties in implementing multi-elements Windkessel and/or non-reflective outlet boundary conditions, incorporate a robust viscoelastic vessel wall model, curvature of arteries and mass loss in smaller arteries and vessel walls.

An attempt to account for the vessel skin friction more accurately is made in the work by Bessems et al. (2007) where the authors consider differential velocity values near the wall region (a viscous layer) and the central core. Thus, the authors have introduced an additional degree of freedom. This approach can improve the accuracy of the computed skin friction to a certain extend but is less accurate than an approach based on the use of the Womersley solution. Another approach to account accurately for the skin friction in a space–time scheme is proposed in Reymond et al. (2009). The authors utilize a waveform obtained at a previous hear-beat cycle, perform the fast Fourier transform (FFT) for every element, build the Womersley solution by computing the Bessel functions of complex argument for every harmonic component, calculate contribution of every harmonic to the skin friction and perform the inverse Fourier transform. For every subsequent cycle, the accuracy of computed skin friction approaches to that of the Womersley solution. This method requires additional computations for every element.

An alternative to space–time method is proposed in Flores et al. (2016), which is based on linearization of the 1D equations and expanding the solution using the Fourier series. In this approach, the problem of wave propagation in an arterial network is solved analytically in the frequency domain, separately for every harmonic component. The pressure and flow rate waveforms are then calculated at any point of the network by numerically computing the inverse Fourier transform to the analytical solution obtained. In this approach, the skin friction can be accurately incorporated via the Womersley solution. Also, the viscoelastic properties of the vessel wall can be included without handling the complexities of the governing equations (Alastruey et al. 2011). As indicated in Flores et al. (2016), this approach allows one to rapidly investigate the role of individual physical properties of a cardiovascular system subjected to a pulsatile waveform. It is important to note that the Fourier transform and calculations carried out in frequency domain are the natural ways for dealing with the wave phenomena. Such important concepts as phase and group velocities can be employed in these methods to explain the distinctive features of wave the pulse propagation and reflection. The frequency domain approach has been successfully used in Sazonov et al. (2017) for developing a non-invasive, aortic aneurysm detection method using a waveform analysis.

Although the method proposed in Flores et al. (2016) is an excellent progress in terms of speed, it has many restrictions. To obtain an analytical solution using this method, every vessel (or its segment between two junctions) is approximated by a cylindrical pipe of constant cross section. Thus, the solution obtained is not general for realistic tapering vessels, found in arterial networks. Another restriction is that the linear algebraic equations have been derived manually for solving the linear problem and thus the method is not easy to employ on an arbitrary arterial network. Finally, the nonlinear effects are not considered by Flores et al. (2016). Therefore, despite its advantages, it is not competitive against the existing 1D modelling methods that use computational algorithms, especially in terms of robustness and accuracy.

In the present work, we propose the generalization of the FFT method by developing an effective and accurate procedure for solving the equations for a tapering vessel or vessel with abnormalities such as aneurysm and stenosis. Also, we account for nonlinear terms in the 1D equations through a nonlinear correction to the linear solution. In this way, we can capture the effect of increasing pulse wave velocity (PWV) downstream as well as steepening of the pulse front. In addition, in the implementation of the proposed method, the system of algebraic equations for every harmonic component is built automatically for any arbitrary arterial network. Finally, the nonlinear equations we employ contain the correct convection and diffusion coefficients.

This paper is organized into the following sections. In Sect. 2, the governing 1D arterial network equations are presented and the nonlinear and viscous terms in these equations are analyzed. Here, different constitutive relations are examined and compared. In Sect. 3, the proposed perturbation method is described, which reduces the nonlinear equations to a set of linear partial differential equations (PDE) which, in turn, are reduced to the ordinary differentials equations (ODE) by the Fourier transform method. An effective method for integration of the derived ODEs for an arbitrary tapering vessel is presented in Sect. 4 and generalized to rapid geometrical variations in vessels in the same section. In Sect. 5, the boundary condition required at inlet, outlet and vessel junctions are described. Here also the method for obtaining a linearized solution for an arbitrary arterial network is explained. In Sect. 6, a method for computing the second-order nonlinear corrections is presented for a single tapering vessel, for the boundary conditions and for full solution in an arterial network. Section 7 compares the present numerical results to those obtained by established 1D numerical methods and experimental results. In the final section, we outline the advantages and future prospects of the proposed approach. Some auxiliary material is presented in Appendix, including generalization of the Womersley solution for flow in a flexible pipe (“Appendix 1”).

## Governing equations for 1D arterial network

The following system of partial differential equations (PDEs) for an arterial network can be derived from the incompressible Navier–Stokes equations (Formaggia et al. 2001; Sherwin et al. 2003)

1 $$\begin{aligned} A_{t}+q_{x}&=0 \end{aligned}$$

2 $$\begin{aligned} q_{t}+\left( \alpha \, \frac{q^{2}}{A}\right) _{\!\!x}+\frac{A}{\rho }\,p_{x}+2\pi \gamma \nu \, \frac{q}{A}&=0 \end{aligned}$$

3 $$\begin{aligned} A&=A(p) . \end{aligned}$$

Here *p*(*x*, *t*) is the pressure; *q*(*x*, *t*) is the flow rate; *A*(*x*, *t*) is the lumen cross-sectional area; subscripts *t* and *x* denote partial derivatives with respect to time *t* and axial coordinate *x*, respectively; $\rho$ and $\nu$ are the blood density and kinematic viscosity, respectively; $\alpha$ and $\gamma$ are dimensionless coefficients defined and discussed in Secs. 2.1 and 2.2. Various forms of the constitutive relations $A = A(p)$ are considered in Sect. 2.3.

Equations (1)–(3) form a basis for the 1D blood flow modelling. Note that these equations do not account for losses in the vessel wall due to its viscoelastic properties and also for blood flow rate losses in small lateral arterial vessels. In the following subsections we make some remarks on nonlinear and viscous terms in the governing PDEs.

### The $\alpha$ parameter

The $\alpha$ coefficient is a factor in the nonlinear convection term in (2) and is defined as

4 $$\begin{aligned} \alpha =\frac{\overline{u^{2}}}{\left( {\bar{u}}\right) ^{2}}=\frac{\frac{1}{A} \int _{S}u^{2}\,{\mathrm {d}}S}{{\bar{u}}^{2}},\qquad {\bar{u}}=\frac{1}{A} \int _{S}u\,{\mathrm {d}}S \end{aligned}$$

where *u* is the flow velocity profile; the integral is taken over the lumen cross section *S*. The $\alpha$ coefficient is called the Coriolis parameter in Formaggia et al. (2001) and is also known as Boussinesq coefficient (Simakov and Kholodov 2009). Its value is assumed to be unity in most of existing models (Mynard and Nithiarasu 2008). This is actually valid for a flow with a uniform velocity profile $u\equiv {\bar{u}}$, which has an infinite velocity gradient at the wall and, hence, the infinite value of the wall shear stress (WSS). Note that as shown in Sherwin et al. (2003), only for $\alpha =1$, (1)–(2) can be rewritten in terms of the mean velocity ${\bar{u}} = q/A$ as

5 $$\begin{aligned} A_{t}+(A{\bar{u}})_{x}&=0 \end{aligned}$$

6 $$\begin{aligned} {\bar{u}}_{t}+ {\bar{u}}{\bar{u}}_{x}+\frac{1}{\rho }p_{x}+\frac{2\pi \gamma \nu }{A}{\bar{u}}&=0 \end{aligned}$$

which are mainly used in the numerical modelling works (eg., Mynard and Nithiarasu 2008; Gamilov et al. 2014). A steady-state flow in a cylindrical blood vessel of constant radius $a=\sqrt{A/\pi }$, i.e., Poiseuille flow, has the parabolic velocity profile of $u(r)=2{\bar{u}}\,(1-r^{2}/a^{2})$. This profile gives $\alpha =4/3$ as shown in Vignon and Taylor (2004). In a pulsating flow, the instant velocity profile varies with time and so should the $\alpha$ parameter (see Formaggia et al. 2003). An effective value of 1.1 for the $\alpha$ parameter is estimated in Smith et al. (2002) and it is used, for example, in Alastruey et al. (2012a).

### Friction coefficient $\gamma$

The friction coefficient $\gamma$ for a cylindrical vessel with lumen radius of *a* can be calculated via the equation

7 $$\begin{aligned} \gamma =\frac{a|u_{r}(a)|}{{\bar{u}}}. \end{aligned}$$

Here, subscript *r* denotes the partial derivative with respect to *r*, which is the polar coordinate in the lumen cross section *S* ($r\in [0,a]$). A uniform velocity profile gives $\gamma =\infty$ as $u_{r}(a)=\infty$.

For the Poiseuille flow $u_{r}(a)=4{\bar{u}}/a$, the $\gamma$ parameter takes a value of 4, which is used in most of the works (e.g. Mynard and Nithiarasu 2008). However, a pulsating flow is characterized by the high near-wall velocity gradient $u_{r}(a)$ much higher than in the steady-state flow. Therefore an effective value of the $\gamma$ coefficient, higher than four is used as well. For example, a value of 11 for $\gamma$ is used in Alastruey et al. (2012a, b). This value is calculated in Smith et al. (2002) by fitting experimental data presented in Hunter (1972).

**Fig. 1:**
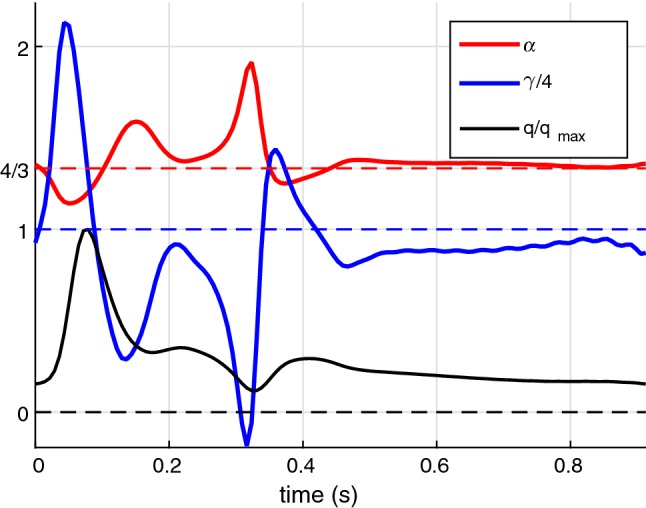
Variation of the nonlinearity coefficient $\alpha$ (red) and the friction coefficient $\gamma$ (divided by 4) (blue) over a cardiac cycle. Normalized flow rate waveform is shown in black. These results are computed using the 3D modelling of the flow in a Carotid artery described in Sazonov et al. (2011)

Analysis based on the Womersley solution (Womersley 1955) shows that the near-wall velocity gradient varies in time and can be higher or lower than that in the steady-state flow. Examples of time-variation of the $\alpha$ and $\gamma$ coefficients are shown in Fig. 1. They are computed using the 3D modelling of the flow in a Carotid artery described in Sazonov et al. (2011). The nonlinearity coefficient $\alpha$ varies around a value of 4/3 and drops down to the value close to 1.1 only in vicinity the waveform peak. The instantaneous value of the friction coefficient $\gamma$ has a larger range as shown in Fig. 1 and it reaches negative values under flow reversal conditions.

From these results we can conclude that assuming coefficients $\alpha$ and $\gamma$ to be constant is very approximate. Assumption of an $\alpha$ coefficient for an uniform velocity profile and the $\gamma$ coefficient for the Poiseuille velocity profile in majority of existing studies should be changed to represent a more accurate pulsatile nature of the flow. A more rigorous approach can be based on convolution with functions $\alpha (t)$ and $\gamma (t)$ in the corresponding terms of the equations. These functions can be determined from the Womersley solution. However, this would complicate tremendously the space–time numerical schemes employed in the 1D modelling approach. Due to the reasons stated above, the nonlinear and viscous terms used in (2) by many of the space–time approaches are not very accurate. However, proposed method can easily incorporate different forms of convection and diffusion coefficients.

### The constitutive relation

Now we consider the first term in (1). Applying the chain rule we can rewrite (1) in the form

8 $$\begin{aligned} \frac{{\mathrm {d}}A}{{\mathrm {d}}p}\,p_{t}+q_{x}=0. \end{aligned}$$

From here one can see that unless *A*(*p*) is a linear function, the first term is nonlinear with respect to *p*. Derivative ${{\mathrm {d}}A}/{{\mathrm {d}}p}\equiv A'(p)$ can be referred to as the cross-sectional vessel compliance. The following dependence *p*(*A*) is proposed in Formaggia et al. (2001) and Sherwin et al. (2003)

9 $$\begin{aligned} p(A)=p_0+\beta \left( \sqrt{A}-\sqrt{A_{0}}\right) . \end{aligned}$$

Here $p_0$ is the external/equilibrium pressure; $A_0$ is the lumen cross-sectional area at equilibrium state $(p, q) = (p_0, 0)$Sherwin et al. (2003); $\beta = \sqrt{\pi }E'h/A$ is a coefficient proportional to the vessel wall stiffness $E'h$ with *h* being the wall thickness and $E^{\prime }=E/(1-\sigma ^{2})$ being the plate/shell analogue of the Young’s modulus and $\sigma$ is the Poisson ratio. For an incompressible material $\sigma =0.5$ and therefore $E' = \frac{4}{3} E$. In Eq. (9), the $\beta$ parameter is assumed to be independent of *A*, which is valid if the vessel wall stiffness $hE'$ is assumed to be independent of strain.

Resolving relation (9) with respect to *A* we see that function *A*(*p*) is obviously nonlinear, i.e.,

10 $$\begin{aligned} A=\left( \sqrt{A_{0}}+(p_0 - p)/{\beta }\right) ^{2}. \end{aligned}$$

Some other dependencies of *p*(*A*) are considered below, and they are also nonlinear. Nevertheless, all of them can be expanded around the equilibrium state: $\varDelta p=p-p_{0}$:

11 $$\begin{aligned} A\approx A_{0}+A_{0}^{\prime }\varDelta p+\frac{1}{2}A_{0}^{\prime \prime }\varDelta p^{2}+\cdots \end{aligned}$$

where prime denotes the derivative with respect to *p* and subscript 0 means that the value is calculated at the equilibrium state. In order to further analyse pressure–area relationship, lets introduce the pulse wave velocity (PWV) of a small perturbation to the equilibrium state $c_0$ as

12 $$\begin{aligned} c_0^2 =\frac{A_0}{\rho A'_0} = \frac{E'_0h_0}{2\rho a_0} \end{aligned}$$

where $a = \sqrt{A/\pi }$ is the lumen radius and subscript 0 denotes that the value is taken at the equilibrium state. Now, the cross-sectional vessel compliance can be represented using the following expansion

13 $$\begin{aligned} \frac{{\mathrm {d}}A}{{\mathrm {d}}p}\equiv A' \approx A_{0}^{\prime }\left[ 1 +\delta _{1}{\hat{p}} +\delta _{2}{\hat{p}} ^{2} +\frac{\delta _{3}}{2}{\hat{p}} ^{3} +\cdots \right] \end{aligned}$$

where the ratio

14 $$\begin{aligned} {\hat{p}} = {\varDelta p}/{(\rho c_0^2)} \end{aligned}$$

is a dimensionless form of pressure perturbation to the equilibrium state and

15 $$\begin{aligned} \delta _{1}=\frac{\rho c_0^2\,A_{0}^{\prime \prime }}{A_{0}},\quad \delta _{2}=\frac{\left( \rho c_0^2\right) ^2 A_{0}^{\prime \prime \prime }}{2A_{0}}, \quad \delta _{3}=\frac{\left( \rho c_0^2\right) ^3 A_{0}^{\prime \prime \prime \prime }}{6A_{0}} ,\ldots \end{aligned}$$

are dimensionless nonlinear coefficients. They are calculated below for several types of constitutive law described in the literature.

*Constant wall stiffness model* Differentiating function (10) and using expansion (13) we obtain

$$\begin{aligned} \delta _{1}= \frac{\rho c_0^2}{\beta \sqrt{A_0}}=\frac{1}{2},\qquad \delta _{2,3,\ldots }=0. \end{aligned}$$

*Olufsen’s model* Model proposed by Olufsen (1999) (used in, eg, in Vignon and Taylor 2004) can be written in the form:

16 $$\begin{aligned} p(A) = p_0+ 2\rho c_0^2 \left( 1 - \sqrt{{A_0}/{A}} \right) . \end{aligned}$$

This model gives

17 $$\begin{aligned} \delta _{1}=\frac{3}{2},\quad \delta _2 = \frac{3}{2},\quad \delta _3 = \frac{15}{4}, \quad \ldots \end{aligned}$$

i.e., threefold increase in nonlinearity coefficient $\delta _1$ compared to the constant stiffness model and additional nonzero coefficients $\delta _2,\delta _3,\ldots$.

*Power law* Both the above models are particular cases for the power law model proposed in Mynard et al. (2010), i.e.

18 $$\begin{aligned} p(A) = p_0 + \frac{2\rho c_0^2}{b}\left( \left( \frac{A_0}{A}\right) ^{b/2} - 1 \right) . \end{aligned}$$

This relationship gives the constant stiffness model (10) for $b=1$ and Olufsen’s model (16) for $b=-1$. For the power law model,

19 $$\begin{aligned} \delta _{1}= \frac{2{-}b}{2},\quad \delta _2 = \frac{(b{-}1)(b{-}2)}{4},\quad \delta _3 = -\frac{(b{-}1)(b{-}2)(3b{-}2)}{8}, \quad \ldots \end{aligned}$$

In Mynard and Smolich (2015) the following formula for the *b* parameter is proposed:

20 $$\begin{aligned} b = {2\rho c_0^2}/{(p_0 - p_{{\mathrm {collapse}}})} \end{aligned}$$

where $p_{{\mathrm {collapse}}}$ is the vessel collapse pressure. For example, for $p_{{\mathrm {collapse}}}=-10\hbox { mmHg}$ (indicated in Mynard and Smolich 2015), the reference pressure $p_0 = 80\hbox { mmHg}$, and for a typical wave velocity in aorta, $c_0 = 5\hbox { m/s}$, we obtain $b = 4.33$, $\delta _1 = -1.17$, $\delta _2 = 1.94$, etc. Note that if *b* parameter obeys Eq. (20) or a similar law, then the nonlinearity parameters $\delta _1,\delta _2,\ldots$ are different in different parts of the arterial system.

In the power law relationship, if $b>1$ then $\delta _1<1/2$ and the vessel wall becomes stiffer when the vessel expands during the pulse propagation. This dependence is indicated by works on arterial wall elastic properties (Holzapfel et al. 2000; Ogden and Saccomandi 2015). If $b=2$ then $\delta _1 = 0$ and the *A*(*p*) dependance becomes linear up to the third-order terms with respect to ${\hat{p}}$. There are other similar models in which the *p*(*A*) dependence is described in terms of elementary functions.

*Armentano et al’s model* One of the earliest models is proposed in Armentano et al. (1995). Here, the lumen diameter, $D = \alpha + \beta \ln p$, where $\alpha$ and $\beta$ are constants. In terms of $p_0,A_0$ and $c_0$ the pressure-area relationship can be written as:

21 $$\begin{aligned} A = A_0\left( 1 + \frac{p_0}{2\rho c_0}\ln \frac{p}{p_0} \right) ^2, \end{aligned}$$

which gives

22 $$\begin{aligned} \delta _{1}=\frac{1}{2} - \frac{\rho c_0^2}{p_0},\quad \delta _2 = \frac{\rho ^2 c_0^4}{p_0^2} - \frac{3}{4}\frac{\rho c_0^2}{p_0} ,\ldots \end{aligned}$$

As observed, the nonlinearity coefficients in the above equation are not constant. They can be very large for small arteries with high wave velocity.

*Kholodov’s model* An exponential/log dependence is proposed in Kholodov (2001) and employed in Gamilov et al. (2014), Simakov and Kholodov (2009), Vassilevski et al. (2015). For $A\ge A_0$ the dependence is exponential and is described by

23 $$\begin{aligned} p = p_0 + \rho c_0^2 \left( \exp \{{A}/{A_0}-1\}-1\right) , \end{aligned}$$

which gives

$$\begin{aligned} \delta _{1}=-1,\quad \delta _{2}=1,\quad \delta _{3}=-3, \ldots \end{aligned}$$

Here, $\delta _1$ is negative and $\delta _2,\delta _3,\ldots$ are nonzero.

It appears that not all studies use identical pressure-area relationships, and the nonlinear parameter $\delta _1$ varies from positive to negative values depending on the model used. These variations between studies indicate that the accuracy of the nonlinear part of the dependence *p*(*A*) is not well established and needs further investigations. Computational results of different studies can agree with each other only if nonlinear effects are small. A small nonlinear term in the *A*(*p*) dependence can be accounted through a correction to the linear problem. Thus, instead of the detailed study on dependance *A*(*p*) or *p*(*A*), one should focus only on nonlinear coefficients $\delta _n$, especially $\delta _1$, in large arteries, where pressure and area variations during the heart cycle are relatively large.

Note that integrating expansion (13), we obtain the useful relationship

24 $$\begin{aligned} \frac{\varDelta A}{A_0} = \frac{\varDelta p}{\rho c_0^2} + \frac{\delta _1}{2} {\hat{p}}^2 + \frac{\delta _2}{3} {\hat{p}}^3 + \cdots , \quad \varDelta A = A - A_0. \end{aligned}$$

This indicates that the pressure and area amplitude parameters ${\hat{p}} = {\varDelta p}/{\rho c_0^2}$ and ${\hat{A}}=\varDelta A/A_0$ are equivalent in the linear approximation.

### Matching conditions at vessel junctions

At vessel junctions we enforce the law of mass conservation, i.e., the flow rate at the outlet of the parent vessel segment, $q_s$, is equal to the sum of the inlet flow rates for the daughter vessel segments, $q_{d}$. The mass conservation at vessel junctions may be written as

25 $$\begin{aligned} q_s = \sum _{d\in {\mathfrak {D}}} q_{d} \end{aligned}$$

where ${\mathfrak {D}}$ is the set of daughter vessel segments connected to parent segment *s*. Equation (25) is linear.

The continuity of the momentum flux requires that the total pressure $p+\frac{1}{2} \rho {\bar{u}}^2$ should be continuous at vessel junctions (Sherwin et al. 2003), i.e.

26 $$\begin{aligned} p_s+\frac{1}{2} \rho {\bar{u}}_s^2 = p_{d}+\frac{1}{2} \rho {\bar{u}}_{d}^2,\quad d\in {\mathfrak {D}}. \end{aligned}$$

This is the matching condition used by majority of 1D arterial network models (Sherwin et al. 2003; Matthys et al. 2007; Mynard and Nithiarasu 2008; Marchandise et al. 2009; Alastruey et al. 2009) and it is nonlinear. Thus, the vessel junction matching condition is the third source of nonlinearity of the problem in addition to the convection term and pressure-area relationship. It is also important to note that some authors apply the continuity of the static pressure *p*, rather than the total pressure as the matching condition (Olufsen 1999; Reymond et al. 2009). A correction to the dynamic part of the pressure in the matching condition which depends on the angle between the parent and daughter vessels is discussed in Formaggia et al. (2003). The possibility to account for an additional pressure loss at junction is considered in Mynard and Valen-Sendstad (2015). This additional term, responsible for the pressure loss in the matching condition, is nonlinear with respect to velocity.

In summary, we can conclude that there are significant differences between studies in dealing with nonlinearity and often the nonlinearity is not satisfactorily represented. These variations are acceptable as long as the effect of nonlinearity is small. Since majority of the existing computational models assume limited nonlinearity in the arteries, we believe that a linearized perturbation method with nonlinear correction term is suitable for solving blood flow equations in a human arterial network.

## Reduction of governing nonlinear PDEs to linear ODEs

### Perturbation method

Multiplying (2) by $\rho /A_0$ and using the conservation mass equation (8) and also swapping the equations we can rewrite them in the form

$$\begin{aligned}&\frac{A}{A_0}p_x + \frac{\rho }{A_0}q_t+\frac{\rho }{A_0}\left( \alpha \frac{q^2}{A}\right) _x + \frac{2\pi \mu \gamma }{A A_0}q =0 \nonumber \\&\quad A' p_t + q_x =0 \end{aligned}$$

where $\mu = \nu \rho$ is the dynamic viscosity. Substituting $A = A_0+\varDelta A$, $A' = A'_0+\varDelta A'$ into these equation and performing some manipulations we obtain

$$\begin{aligned}&p_x+\frac{\varDelta A}{A_0}p_x + \frac{\rho }{A_0}q_t + \frac{\rho }{A_0}\left( \alpha \frac{q^2}{A}\right) _x + \frac{2\pi \mu \gamma }{A_0^2}q\\&\quad + \frac{2\pi \mu \gamma }{A A_0}- \frac{2\pi \mu \gamma }{A_0^2}q =0\\&\quad q_x + A'_0 p_t + \varDelta A' p_t =0. \end{aligned}$$

Now retaining the linear and linearized terms on the left-hand side and substituting $A_0'=A_0/(\rho c_0^2)$ in virtu of (12), we, respectively, have

27 $$\begin{aligned} p_x + \frac{\rho }{A_0}q_t + \frac{2\pi \mu \gamma }{A_0^2}q&= -\frac{\rho }{A_0}\left( \alpha \frac{q^2}{A}\right) _x - \frac{\varDelta A}{A_0}p_x \nonumber \\&\qquad + \frac{2\pi \mu \gamma }{A_0} \left( \frac{1}{A_0} {-} \frac{1}{A}\right) q \end{aligned}$$

28 $$\begin{aligned} q_x + \frac{A_0}{\rho c_0^2} p_t&=-\varDelta A'\,p_t. \end{aligned}$$

We can rewrite Eqs. (27)–(28) in the matrix form:

29 $$\begin{aligned} {\varvec{L}}{\varvec{u}} = {\varvec{n}} \end{aligned}$$

where ${\varvec{L}}$ is the linear operator, ${\varvec{u}}$ is the state vector, i.e.,

30 $$\begin{aligned} {\varvec{L}} = \begin{bmatrix} \displaystyle \frac{\partial }{\partial x}&\quad \displaystyle \frac{\rho }{A_0}\frac{\partial }{\partial t} + \frac{2\pi \mu \gamma }{A_0^2} \\ \displaystyle \frac{A_0}{\rho c_0^2}\frac{\partial }{\partial t}&\quad \displaystyle \frac{\partial }{\partial x} \end{bmatrix} ,\quad {\varvec{u}} = \begin{bmatrix} \displaystyle p \\ \displaystyle q \end{bmatrix}, \end{aligned}$$

${\varvec{n}}$ is a vector of nonlinear terms:

31 $$\begin{aligned} {\varvec{n}} = \begin{bmatrix} \displaystyle -\frac{\rho }{A_0}\left( \alpha \frac{q^2}{A}\right) _x - \frac{\varDelta A}{A_0}p_x + \frac{2\pi \mu \gamma }{A_0} \left( \frac{1}{A_0} {-} \frac{1}{A}\right) q \\ \displaystyle -\varDelta A'\,p_t \end{bmatrix}. \end{aligned}$$

If the nonlinear terms are small, Eq. (29) can be solved using subsequent approximation (perturbation) method ${\varvec{u}} = {\varvec{u}}^{(1)}+ {\varvec{u}}^{(2)}+ \cdots$, where ${\varvec{u}}^{(m)}$ satisfies the equation

32 $$\begin{aligned} {\varvec{L}}{\varvec{u}}^{(m)} = {\varvec{n}}^{(m)}, \qquad m = 1,2,\ldots \end{aligned}$$

Here ${\varvec{n}}^{(m)}$ are the nonlinear correction terms:

33 $$\begin{aligned}&{\varvec{n}}^{(1)} = \begin{bmatrix} 0 \\ 0 \end{bmatrix},\nonumber \\&\quad {\varvec{n}}^{(2)} = \begin{bmatrix}\displaystyle -\frac{\rho }{A_0}\left( \alpha ^{(1)}\frac{(q^{(1)})^2}{A_0}\right) _x - \frac{1}{\rho c_0^2}\varDelta p^{(1)}p^{(1)}_x + \frac{2\pi \mu \gamma }{\rho c_0^2A_0^2}\varDelta p^{(1)}q^{(1)}\\ \displaystyle -\delta _1\frac{A_0}{\rho ^2 c_0^4}\varDelta p^{(1)}p^{(1)}_t \end{bmatrix}, \ldots \end{aligned}$$

To derive (33) we have employed the following approximations

$$\begin{aligned} \varDelta A&\approx A'_0\varDelta p^{(1)} = \frac{A_0}{\rho c_0^2}\varDelta p^{(1)}\\ \frac{1}{A_0} - \frac{1}{A}&\quad \approx \frac{A'_0}{A_0^2}\varDelta p^{(1)} = \frac{1}{A_0\rho c_0^2}\varDelta p^{(1)}\\ \varDelta A'&\quad \approx A'_0\delta _1 \frac{\varDelta p^{(1)}}{\rho c_0^2} = \delta _1\frac{A_0}{2\rho ^2 c_0^4}\varDelta p^{(1)}. \end{aligned}$$

Equation (32) is linear for every *m*. It is homogeneous for $m=1$. The right-hand side for $m>1$ depends on $\varDelta p^{(m')},q^{(m')}$ calculated at previous iterations: $m'<m$:

$$\begin{aligned} {\varvec{L}}{\varvec{u}}^{(1)}&= {\varvec{0}} \\ {\varvec{L}}{\varvec{u}}^{(2)}&= {\varvec{n}}^{(1)}\left( {\varvec{u}}^{(1)}\right) \\ {\varvec{L}}{\varvec{u}}^{(3)}&= {\varvec{n}}^{(2)}\left( {\varvec{u}}^{(1)},{\varvec{u}}^{(2)}\right) \\&\cdots \end{aligned}$$

### The Fourier transform method

The real heartbeat waveform is quasi-periodic, but the numerical simulations assume periodicity for convenience. Often simulations are repeated over several cardiac cycles before the results are used to avoid the effect of any initial conditions (Mynard and Nithiarasu 2008). Periodicity may typically be expressed as

$$\begin{aligned} p(t + T) = p(t)\quad {\text {and}}\qquad q(t + T) = q(t). \end{aligned}$$

In this case the Fourier series expansion is an appropriate method for solving the partial differential equations (PDE), i.e.,

34 $$\begin{aligned} \begin{bmatrix} p\\q \end{bmatrix} = \sum _{n=-\infty }^{+\infty } \begin{bmatrix} P_n\\Q_n \end{bmatrix} {\mathrm{e}}^{{i}\omega _n t} \end{aligned}$$

where *i* is the imaginary unity,

$$\begin{aligned} \omega _n = \frac{2\pi }{T}n\quad {\text {with}} \qquad n = 0,\pm \,1, \pm \,2, \ldots \end{aligned}$$

is the frequency of *n*th harmonic component and $P_n(x)$ and $Q_n(x)$, respectively, represent the *n*th harmonic component (complex amplitudes of a harmonic wave at current *x*). They can be calculated using the integral over the cardiac period *T*, which is the direct Fourier transform of a periodic function, i.e.,

35 $$\begin{aligned} \begin{bmatrix} P_n(x)\\Q_n(x) \end{bmatrix} = \frac{1}{T}\int _{0}^{T} \begin{bmatrix} p(x,t)\\q(x,t) \end{bmatrix} {\mathrm{e}}^{-{i}\omega _n t}{\mathrm {d}}t. \end{aligned}$$

Applying the Fourier transform (35) to Eq. (32), we obtain a system of ordinary differential equations (ODE):

36 $$\begin{aligned} \varvec{{\tilde{L}}}_n{\varvec{U}}^{(m)}_n = \varvec{{N}}^{(m)}_n, \qquad m = 1,2,\ldots \end{aligned}$$

where ${\varvec{U}}^{(m)}_n = [P^{(m)}_n, Q^{(m)}_n]^{\mathrm{T}}$ is the *n*th harmonic component of the state vector ${\varvec{u}}^{(m)}$. The linear operator contains only full derivatives, i.e.,

37 $$\begin{aligned} \varvec{{\tilde{L}}}_n = \begin{bmatrix} \displaystyle \frac{{\mathrm {d}} }{{\mathrm {d}} x}&\displaystyle \frac{{i}\omega _n\rho }{A_0} + \frac{2\pi \mu \gamma }{A_0^2}\\ \displaystyle \frac{{i}\omega _nA_0}{\rho c_0^2}&\displaystyle \frac{{\mathrm {d}} }{{\mathrm {d}} x} \end{bmatrix}. \end{aligned}$$

The right-hand side of Eq. (36) becomes

38 $$\begin{aligned} {\varvec{N}}^{(1)}_n = \begin{bmatrix}0 \\ 0\end{bmatrix},\qquad {\varvec{N}}^{(2)}_n = \frac{1}{T}\int _{0}^{T} {\varvec{n}}^{(2)} {\mathrm{e}}^{{-i}\omega _n t}{\mathrm {d}}t. \end{aligned}$$

Now, instead of a set of PDEs, we have a set of independent linear ODEs for $n=0,1,2,\ldots ,N$, where *N* is the number of harmonic components required. We do not need to solve these equations for negative *n* as a real ${\varvec{u}}^{(m)}$ results in ${\varvec{U}}^{(m)}_{-n} = \left( {\varvec{U}}^{(m)}_n\right) ^*$, where $*$ denotes a complex conjugate.

## Integration of the ODEs

Since we are working with the basic state values of *A*, *q* and *c* here, we omit subscript 0 in this section and in Sect. 6 as it can be confused with the zeroth harmonic component. In these sections we follow $c=c_{0}$ and $A=A_{0}$ and $q=q_{0}$ and regard them as independent of the wave amplitude. In the subsections below we consider the linear approximation, i.e. calculation of $P_n^{(1)},Q_n^{(1)}$ and the nonlinear corrections are considered in Sect. 6.

### Zeroth harmonic component

In arteries of circular cross section, the flow is Poiseuille in nature and $\gamma =4$. Therefore, ODE (36) for $n=0$ takes the form

$$\begin{aligned} \left( P^{(1)}_0\right) _x+8\pi \mu \frac{Q^{(1)}_0}{A^{2}}&= 0\\ \left( Q^{(1)}_0\right) _x&= 0 \end{aligned}$$

which has the solution

39 $$\begin{aligned} Q^{(1)}_0 = {\bar{q}},\qquad P^{(1)}_0 = {\bar{p}}(0)-8\pi \mu {\bar{q}}\int _0^x\frac{{\mathrm {d}}x}{A^{2}} \end{aligned}$$

where ${\bar{q}}$ and ${\bar{p}}(0)$ are, respectively, the flow rate and the inlet pressure ($x=0$) averaged over a cardiac cycle, i.e.,

$$\begin{aligned} {\bar{q}}= \frac{1}{T}\int _0^T q(t)\,{\mathrm {d}}t, \qquad {\bar{p}}(0)= \frac{1}{T}\int _0^T p(0,t)\,{\mathrm {d}}t. \end{aligned}$$

### Nonzeroth harmonic components

In this section we omit subscript *n* in variables $P_n^{(1)}$, $Q_n^{(1)}$ and $\omega _n$ to make the equations easy to follow. For the linear approximation, we have the following ODEs:

40 $$\begin{aligned} P_{x}^{(1)} + {i}\omega \rho \phi ^{2}\frac{Q^{(1)}}{A}&= 0 \end{aligned}$$

41 $$\begin{aligned} Q_{x}^{(1)}+\frac{{i}\omega A}{\rho c^{2}}P^{(1)}&= 0 \end{aligned}$$

where

42 $$\begin{aligned} \phi (x) =\sqrt{1-{i}\frac{2\pi \nu \gamma }{\omega A}} \end{aligned}$$

is a viscous factor which accounts for wave decays due to viscous losses, $A = A(x)$, $c=c(x)$ and $\gamma = \gamma \left( a(x)\sqrt{-{i}\nu /\omega }\,\right)$. The influence of viscosity on wave propagation and evaluation of $\phi$ is considered in Sect. 4.3. Explicit formulas for $\gamma$ are derived in “Appendix 1” for a flexible cylindrical pipe.

### Vessel of constant cross section and wall stiffness

The ODEs (40)–(41) do not admit an analytical solution in a closed form for a generic tapering vessel. Therefore, we first consider the simplest case of uniform vessel with constant parameters along the vessel axis.

*Solution as a sum of travelling waves* If *A*, *c* and $\gamma$ are constant along the vessel, then we can write a general analytical solution in the linear approximation using the forward, $[P_f,Q_f]^{\mathrm{T}}$, and backward, $[P_b,Q_b]^{\mathrm{T}}$, harmonic travelling waves as particular solutions, i.e.,

43 $$\begin{aligned} \begin{bmatrix} P^{(1)}(x)\\ Q^{(1)}(x) \end{bmatrix}= & C_f \begin{bmatrix} P_f\\ Q_f \end{bmatrix} + C_b \begin{bmatrix} P_b\\ Q_b \end{bmatrix} = C_f \begin{bmatrix} {\mathrm{e}}^{-{i}k x}\\ \tilde{Y} {\mathrm{e}}^{-{i}k x} \end{bmatrix} \nonumber \\&\quad + C_b \begin{bmatrix} {\mathrm{e}}^{+{i}k x}\\ -\tilde{Y} {\mathrm{e}}^{+{i}k x} \end{bmatrix}. \end{aligned}$$

Here $k = \omega \phi /c$ is the wavenumber; $C_f$ and $C_b$ are pressure amplitudes of, respectively, forward and backward harmonic waves; parameter $\tilde{Y} = \displaystyle {{A}/(\rho c \phi )}$ is the characteristic vessel compliance that is an inverse value to the characteristic vessel impedance (Westerhof et al. 2005; Hametner et al. 2013)

44 $$\begin{aligned} \tilde{Z} = \frac{P_f}{Q_f} = \frac{\rho c \phi }{A} = \frac{1}{\tilde{Y}} \end{aligned}$$

i.e. the pressure to flow rate ratio in the forward propagating wave along a uniform pipe. This parameter is frequency dependent in a pipe with viscous losses. This dependence is accounted in the factor $\phi (\omega )$. In solution form (43), the pressure and flow rate fields are fully determined by two numbers: $C_f$ and $C_b$.

*Effect of blood viscosity on wave propagation* In the presence of viscosity, the wavenumber is complex $k = \omega \phi /c$. The real part of $\phi$ affects the wave speed (slowing it down) and increases the total phase incursion ${\mathrm {Re}}\,(kL)$ in the segment of length *L*. Its imaginary part, ${\mathrm {Im}} \phi \le 0$, indicates the wave decay during propagation. The wave decay per unit of length equals to $\exp \{-{\mathrm {Im}} k\}$.

**Fig. 2:**
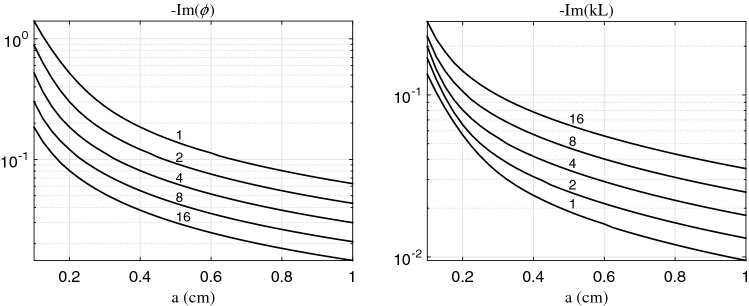
Dependence of the imaginary part of the $\phi$ parameter (left) and the total phase incursion *kL* (right) on lumen radius *a* and frequency *f* (indicated above every curve in Hz). Here $L=10\,\hbox {cm}$ is taken

Dependence of $-{\mathrm {Im}} (\phi )$ on the lumen radius *a* and the wave frequency $f=\omega /2\pi$ is shown in Fig. 2(left) for $\gamma$ calculated via Eq. (130) (see “Appendix 1”). One can see that its contribution to the wavenumber is essential for narrow vessels and at low frequencies. The wavenumber varies essentially along a tapering vessel at low frequencies. Therefore contribution of viscous losses looks to be more crucial for lower frequencies.

Due to viscous losses, the wave amplitude decays by a factor $\exp \{-{\mathrm {Im}} kL\}$ after propagation through the vessel. Therefore, since the total phase incursion is small for low frequencies, the relatively large imaginary part of the wavenumber does not cause the essential decay of the wave in a vessel. This is indicated in Fig. 2(right) where plots of $-{\mathrm {Im}} kL$ are depicted for $L = 10\,\hbox {cm}$. One can see that the decay at the total length *L* is higher at a higher frequency.

*Transmission matrix* An alternative form of a general solution can be used if the vessel inlet conditions, *P*(0), *Q*(0), are given as

45 $$\begin{aligned} \begin{bmatrix} P(x) \\ Q(x) \end{bmatrix} = {\varvec{T}}(x) \begin{bmatrix} P(0) \\ Q(0) \end{bmatrix} = \begin{bmatrix} \cos kx&\quad -{i}\tilde{Z}\sin kx \\ -{i}\tilde{Y}\sin kx&\quad \cos kx \end{bmatrix} \begin{bmatrix} P(0) \\ Q(0) \end{bmatrix} \end{aligned}$$

Here ${\varvec{T}}(x)$ is the transmission matrix, which is equal to the identity matrix ${\varvec{I}}$ at $x=0$:

46 $$\begin{aligned} {\varvec{T}}(0) = {\varvec{I}} = \begin{bmatrix} 1&\quad 0 \\ 0&\quad 1 \end{bmatrix}. \end{aligned}$$

In this approach, the pressure and flow rate fields are determined by two numbers *P*(0) and *Q*(0). Note that in Flores et al. (2016), two numbers: *P*(0) and *P*(*L*) are selected to determine the total field in the vessel. Below we show that the choice of *P*(0) and *Q*(0) has some advantages for numerical implementation.

### Tapering vessel

ODEs (40) and (41) do not admit a closed from analytical solution for arbitrary functions *A*(*x*) and *c*(*x*). After analysing different possible approaches, we have selected the following two approaches.

*Numerical integration of initial value problems* ODEs (40) and (41) can be integrated numerically. To compute entries of transmission matrix ${\varvec{T}}$, we should integrate ODEs (40) and (41) twice with the initial conditions, i.e.,

47 $$\begin{aligned} \begin{bmatrix}P^{(1)}(0)\\Q^{(1)}(0)\end{bmatrix} = \begin{bmatrix}1\\0\end{bmatrix}\qquad {\text{ and }}\qquad \begin{bmatrix}P^{(1)}(0)\\Q^{(1)}(0)\end{bmatrix} = \begin{bmatrix}0\\1\end{bmatrix}. \end{aligned}$$

The solution to the first initial value problem gives matrix elements $T_{11}$ and $T_{21}$ and the solution to the second initial value problem gives matrix elements $T_{12}$ and $T_{22}$. This method is accurate and effective for the lowest harmonic components but looses its speed and accuracy for high-frequency harmonic components as the solution becomes fast oscillating.

*Piecewise conic approximation* Eliminating *Q* from ODEs (40) and (41), we obtain the second-order ODE with respect to *P*. After obtaining a solution, we can calculate *Q* as

48 $$\begin{aligned} P_{xx} + 2\left( \frac{a_x}{a}-\frac{\phi _x}{\phi }\right) P_{x}+k^2P=0, \qquad Q = -\frac{{\tilde{Y}}}{{i}k}P_x. \end{aligned}$$

Note that wave velocity *c* varies along the vessel approximately proportional to $a^{-1/2}$, i.e. slower than the lumen radius. The variable $\phi$ varies fast only in a narrow and strongly tapering vessel. If we approximate *c*, $\phi$ and $k=\omega \phi /c$ by their averaged values, we have

49 $$\begin{aligned} k\approx {\bar{k}}= & \frac{1}{L}\int _0^L k(x)\,{\mathrm {d}}x \quad c\approx {\bar{c}} = \frac{1}{L}\int _0^L c(x)\,{\mathrm {d}}x \nonumber \\ \phi \approx {\bar{\phi }}= & \frac{1}{L}\int _0^L \phi (x)\,{\mathrm {d}}x. \end{aligned}$$

Now ODE (48) admits an analytical solution in a number of cases. We consider here the case of a conic vessel segment: $a(x)=a_1 + (a_2-a_1)(x/L)$, then we can write the general solution in the form of forward and backward waves in which the pressure amplitude grows toward the narrower edge of the vessel, i.e.,

50 $$\begin{aligned} \begin{bmatrix} P^{(1)}(x)\\ Q^{(1)}(x) \end{bmatrix}= & C_f \frac{a_1}{a}\begin{bmatrix} \displaystyle {\mathrm{e}}^{-{i}{\bar{k}} x}\\ \displaystyle \tilde{Y}\left( 1 - {i}\zeta \right) {\mathrm{e}}^{-{i}{\bar{k}} x} \end{bmatrix}\nonumber \\&\quad + C_b \frac{a_1}{a}\begin{bmatrix} \displaystyle {\mathrm{e}}^{+{i}{\bar{k}} x}\\ \displaystyle \tilde{Y}\left( {-}1 - {i}\zeta \right) {\mathrm{e}}^{+{i}{\bar{k}} x} \end{bmatrix} \end{aligned}$$

where

51 $$\begin{aligned}&\tilde{Y}(x) = \frac{\pi a^2(x)}{\rho {\bar{c}}{\bar{\phi }}}=\frac{1}{\tilde{Z}(x)},\quad \zeta (x) = \frac{a_x}{{\bar{k}}a(x)} ,\nonumber \\&\quad a_x = \frac{a_2-a_1}{L} = \hbox {const}. \end{aligned}$$

The transmission matrix for this vessel is

52 $$\begin{aligned} {\varvec{T}}(x)= \begin{bmatrix} \displaystyle \frac{a_{1}}{a}\cos {\bar{k}}x+\zeta \sin {\bar{k}}x&\displaystyle -{i}\tilde{Z}_{1}\frac{a_{1}}{a}\sin {\bar{k}}x \\ \displaystyle -{i}\tilde{Y}_{1}\frac{a}{a_{1}}\left[ (1+\zeta _{1}\zeta )\sin {\bar{k}}x+(\zeta -\zeta _{1})\cos {\bar{k}}x\right]&\quad \displaystyle \frac{a}{a_{1}}\cos {\bar{k}}x-\zeta _{1}\sin {\bar{k}}x \end{bmatrix} \end{aligned}$$

where $$\tilde{Y}_1 = \tilde{Y}(0)$$, $$\tilde{Z}_1 = \tilde{Z}(0)$$, $$\zeta _1 = \zeta (0)$$.

A tapering vessel can be approximated by a sequence of truncated conic elements with values $c,\phi$ and *k*, approximated by their average value over every element. Let $\{x_0=0,x_1,\ldots ,x_{{N_e}-1},x_{{N_e}}=L\}$ be edge points of the cones. Here, ${N_e}$ is the number of conic elements.

To accelerate the computation, the inlet and outlet values of every *i*th element can be used to calculate the corresponding mean values:

53 $$\begin{aligned}&{\bar{k}}_i \approx \frac{\omega }{2} \left( \frac{\phi _{i-1}}{c_{i-1}}+\frac{\phi _{i}}{c_{i}}\right) , \quad {\bar{c}}_i \approx \frac{1}{2} \left( c_{i-1} + c_{i}\right) ,\nonumber \\&\quad {\text {and}}\quad {\bar{\phi }}_i \approx \frac{1}{2} \left( \phi _{i-1} + \phi _{i}\right) \end{aligned}$$

where subscript *i* indicates that the value is calculated at point $x_i$. Using (52) we can write the transmission matrix ${\varvec{T}}_i$ of every element as

54 $$\begin{aligned} {\varvec{T}}_{i}= \begin{bmatrix} \displaystyle \frac{a_{i-1}}{a_{i}}\cos \theta _{i}+\zeta _{i}\sin \theta _{i}&\displaystyle -{i}{\bar{Z}}_{i}\sin \theta _{i} \\ \displaystyle -{i}{\bar{Y}}_{i}\left[ (1+\zeta _{i-1}\zeta _{i})\sin \theta _{i}+(\zeta _{i}-\zeta _{i-1})\cos \theta _{i}\right]&\displaystyle \frac{a_{i} }{a_{i-1}}(\cos \theta _{i}-\zeta _{i}\sin \theta _{i}) \end{bmatrix}. \end{aligned}$$

Here,

$$\begin{aligned}&\theta _{i}={\bar{k}}_{i}h_{i},\quad h_{i}=x_{i}-x_{i-1},\quad \zeta _{i}=\frac{a_{xi}}{{\bar{k}}_{i}a_{i}},\quad a_{xi}=\frac{a_{i}-a_{i-1}}{h_{i}},\\&\quad {\text {and}}\quad {\bar{Y}}_{i}=\frac{\pi a_{i-1}a_{i}}{\rho {\bar{c}}_{i}\bar{\phi _{i}}}=\frac{1}{{\bar{Z}}_{i}} \end{aligned}$$

where $a_{i}$ is the lumen radius at $x_{i}$. The transmission matrix ${\varvec{T}}$ of total segment can be calculated through the product of transmission matrices ${\varvec{T}}_i$ for every element as:

55 $$\begin{aligned} {\varvec{T}} = {\varvec{T}}_{{N_e}}\,\cdots {\varvec{T}}_2\,{\varvec{T}}_1 . \end{aligned}$$

*Accuracy of the piecewise conic approximation* To study the accuracy of the piecewise conic approximation, we compute the reflection $R(\omega )$ and transmission $S(\omega )$ coefficients of a tapering vessel, located between two cylindrical pipes with matched areas and wave velocities. The Right Carotid segment has been selected from the arterial network described in Mynard and Nithiarasu (2008) having simultaneously the largest length and highest lumen radius gradient: the length is $L=9.4\hbox { cm}$, the inlet and outlet diameters, respectively, are $D_1 = 6.75\hbox { mm}$ and $D_2 = 3.5\hbox { mm}$. The vessel is approximated by a cone. The wave velocity dependence on lumen radius is taken to be approximated by Eq. (102) proposed in Blanco et al. (2015).

Three basic element lengths are selected $h_b=2,1$ and $0.5\hbox { cm}$ for the conic approximation. These lengths are adjusted to the vessel length by the rule: ${N_e} = \lceil L/h_b\rceil$, $h = L/{N_e}$, where $\lceil x\rceil$ means the closest integer equal or greater than *x*. Therefore the element lengths are adjusted to values $h\approx 1.88,0.94$ and $0.49\hbox { cm}$.

**Fig. 3:**
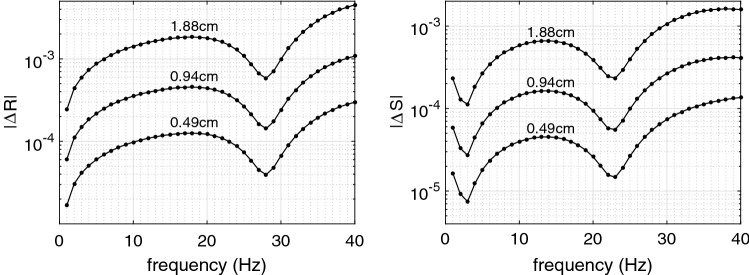
Accuracy of the reflection coefficient $\varDelta R = |R_{\mathrm {numer}}-R_{\mathrm {conic}}|$ (left) and transmission coefficient $\varDelta S = |S_{\mathrm {numer}}-S_{\mathrm {conic}}|$ versus frequency *f* for different element lengths *h* (indicated above every curve)

The reflection $R_{\mathrm {conic}}$ and transmission $S_{\mathrm {conic}}$ coefficients computed in the conic approximation are compared with the same coefficients $R_{\mathrm {numer}}$, $S_{\mathrm {numer}}$ computed by the numerical integration with the accuracy of $10^{-10}$. Results of comparison are presented in Fig. 3. Observe that the accuracy is very good, especially for the first five harmonic components containing more than 90% of the pulse wave energy.

## Boundary conditions

Consider a network containing $N_s$ vessels and vessel segments with one inlet segment $s=1$ and a number of terminating segments. In every segment the axial coordinate *x* is referenced from its inlet. The flow parameters $P_{ns}(x) = P_s(x;\omega _n)$ and $Q_{ns}(x) = Q_s(x;\omega _n)$ in the *s*th segment can be calculated by the methods described above if the inlet flow parameters $P_{ns}(0)$ and $Q_{ns}(0)$ are known.

Introduce the notations ${\hat{P}}_{ns}=P_{ns}(0)$ and ${\hat{Q}}_{ns}=Q_{ns}(0)$, where $s=1,\ldots ,N_s$. Thus ${\hat{P}}_{ns},{\hat{Q}}_{ns}$ are inlet amplitudes for every segment of the network. To compute the flow in the entire network, we have first to calculate $2N_s$ unknowns $[{\hat{P}}_{n1},{\hat{Q}}_{n1},\ldots , {\hat{P}}_{nN_s},{\hat{Q}}_{nN_s}]$, for every harmonic component. Hence, we have to derive $2N_s$ equations with respect to these unknowns from the inlet, junction and outlet boundary conditions.

In Flores et al. (2016) the following $2N_s$ unknowns are taken: $[P_{n1}(0),P_{n1}(L_1),\ldots ,P_{nN_s}(0),P_{nN_s}(L_{N_s})]$. This is suitable if the vessels are approximated by uniform pipes without nonlinear correction. In stead of dealing with $P_{ns}(0)$ and $Q_{ns}(0)$, we can actively use the transmission matrices ${\varvec{T}}$ to calculate solutions for tapering vessels. Also, this approach simplifies computation of nonlinear corrections.

*Inlet boundary condition* Let the periodic pressure waveform is set at the inlet of the network $p^{\mathrm {in}}(t)=p^{\mathrm {in}}(t+T)$ . We regard $p^{\mathrm {in}}(t)$ as a full pressure taken from direct measurements (i.e. sum of forward and the backward waves). Then the boundary conditions is

56 $$\begin{aligned} {\hat{P}}_{n1} = P_n^{\mathrm {in}}, \qquad n = 0,1,\ldots ,N. \end{aligned}$$

where $P_n^{\mathrm {in}}$ are calculated via the Fourier transform (35).

If $p^{\mathrm {in}}(t)$ is a forward propagating waveform (as it used in Mynard and Nithiarasu (2008) as an inlet boundary condition), then we can rewrite solution (50) in the form

57 $$\begin{aligned}&\begin{bmatrix} {\hat{P}}_{n1} \\ {\hat{Q}}_{n1} \end{bmatrix} = P^{\mathrm {in}}_n \begin{bmatrix} 1 \\ \tilde{Y}_{n1}(0)(1-{i}\zeta _{n1}(0)) \end{bmatrix}\nonumber \\&\qquad \qquad + C_b \begin{bmatrix} 1 \\ \tilde{Y}_{n1}(0)(-1-{i}\zeta _{n1}(0)) \end{bmatrix} \end{aligned}$$

where $\tilde{Y}_{n1}(0)$ and $\zeta _{n1}(0)$ are inlet parameters of the first element in the first segment. Now eliminating the amplitude of the backward wave $C_b$, we obtain an equation with respect to unknowns ${\hat{P}}_{n1}$ and ${\hat{Q}}_{n1}$. Substituting ${\hat{P}}_{n1}$ and ${\hat{Q}}_{n1}$, we have

58 $$\begin{aligned} \left( 1+{i}\zeta _{n1}(0) \right) {\hat{P}}_{n1} + \tilde{Z}_{n1}(0) {\hat{Q}}_{n1} = 2\,P_n^{\mathrm {in}}, \quad n>0 \end{aligned}$$

where $\tilde{Z}_{n1}(0)$ is the characteristic impedance of very first element of the first segment.

Analogously, if the periodic flow rate waveform $q^{\mathrm {in}}(t)=q^{\mathrm {in}}(t+T)$ is given at the inlet and it is the full waveform then the inlet boundary condition is

59 $$\begin{aligned} {\hat{Q}}_{n1} = Q_n^{\mathrm {in}}, \quad n=0,1,\ldots ,N. \end{aligned}$$

where $Q_n^{\mathrm {in}}$ are calculated via the Fourier transform (35).

If $q^{\mathrm {in}}(t)$ is the only forward propagating waveform then for the nonzeroth harmonics, we can derive in the similar manner

60 $$\begin{aligned} \tilde{Y}_{n1}(0)\left( 1+{i}\zeta _{n1}(0) \right) {\hat{P}}_{n1} + {\hat{Q}}_{n1} = 2\,Q_n^{\mathrm {in}}, \quad n>0. \end{aligned}$$

*Matching conditions at junctions* Consider the *s*th segment of length $L_s$ connected to ${\mathcal {D}}$ daughter segments at its outlet. Remind that ${\hat{P}}_{ns}$ and ${\hat{Q}}_{ns}$ are inlet flow parameters for the *s*th segment for the *n*th harmonic component. Now, the outlet flow parameters can be written in the linear approximation through the transmission matrix ${\varvec{T}}_{ns}$ for the *s*th segment as

61 $$\begin{aligned} P_{ns}^{(1)}(L_s) = T_{11}^{ns}{\hat{P}}_{ns} + T_{12}^{ns}{\hat{Q}}_{ns},\qquad {\text {and}}\quad Q_{ns}^{(1)}(L_s) = T_{21}^{ns}{\hat{P}}_{ns} + T_{22}^{ns}{\hat{Q}}_{ns}. \end{aligned}$$

Hereinafter ${\varvec{T}}_{ns}$ without argument means ${\varvec{T}}_{ns} = {\varvec{T}}_{ns}(L_s)$. For the zeroth harmonic relations (61) are different (see solution (39))

62 $$\begin{aligned} Q^{(1)}_{0s}(L_s) = {\hat{Q}}_{0s},\qquad P^{(1)}_{0s}(L_s) = {\hat{P}}_{0s}-8\pi \mu {\hat{Q}}_{0s}\int _0^{L_s}\frac{{\mathrm {d}}x}{A^{2}}. \end{aligned}$$

but they can be written in the form of (61) if we define the transmission matrix for $\omega = 0$, i.e.,

63 $$\begin{aligned} {\varvec{T}}_{0s}(x) = \begin{bmatrix} 1&\quad {-}8\pi \mu \int _0^{x}{\mathrm {d}}x/A_s^{2}(x)\\ 0&1 \end{bmatrix}. \end{aligned}$$

If we denote the set of daughter segments associated with a parent segment *s* as ${\mathfrak {D}}=\{d_1,\ldots ,d_{{\mathcal {D}}} \}$, the continuity of the total pressure and the mass conservation at junction can, respectively, be written as

64 $$\begin{aligned} P_{ns}^{(1)}(L_s) = P_{nd}(0), \quad d\in {\mathfrak {D}}, \qquad {\text {and}}\quad Q_{ns}^{(1)}(L_s) = \sum _{d\in {\mathfrak {D}}} Q_{nd}(0). \end{aligned}$$

Substituting relations (64) into conditions (61), we obtain ${\mathcal {D}}+1$ linear homogeneous equations as

65 $$\begin{aligned} \begin{array}{rcl} T_{11}^{ns}{\hat{P}}_{ns} + T_{12}^{ns}{\hat{Q}}_{ns} - {\hat{P}}_{nd_1} &=& 0\\ &\vdots &\\ T_{11}^{ns}{\hat{P}}_{ns} + T_{12}^{ns}{\hat{Q}}_{ns} - {\hat{P}}_{nd_{{\mathcal {D}}}} &=& 0\\ T_{21}^{ns}{\hat{P}}_{ns} + T_{22}^{ns}{\hat{Q}}_{ns} - {\hat{Q}}_{nd_1} - \cdots - {\hat{Q}}_{nd_{{\mathcal {D}}}} &=& 0. \end{array} \end{aligned}$$

*Matching conditions at junctions with merging arteries* In some junctions two parent arteries can merge to form a single daughter artery. For example, the left and right vertebral arteries merge to form the basilar artery which is a common daughter segment for the vertebral arteries. There are few similar backward bifurcations in the cerebral arterial system. Similar situation can occur in an arterial system with a bypass. In a general case, for the node with ${\mathcal {P}}$ parent segments and ${\mathcal {D}}$ daughter segments, we have the following set of equations

66 $$\begin{aligned}&P_{n{p_1}}^{(1)}(L_1)=\ldots P_{np_{{\mathcal {P}}}}^{(1)}(L_{p_{{\mathcal {P}}}}) = {\hat{P}}_{nd_1} = \ldots ={\hat{P}}_{nd_{{\mathcal {D}}}} \end{aligned}$$

67 $$\begin{aligned}&Q_{n{p_1}}^{(1)}(L_1)+\cdots +Q_{np_{{\mathcal {P}}}}^{(1)}(L_{p_{{\mathcal {P}}}}) = {\hat{Q}}_{nd_1} + \cdots + {\hat{Q}}_{nd_{{\mathcal {D}}}} . \end{aligned}$$

The number of equations for the junction always equals the nodal index of the junction: ${\mathcal {P}}+{\mathcal {D}}$. In the particular case of a junction with ${\mathcal {P}}=2, {\mathcal {D}}=1$ occurring among the cerebral arteries gives us three equations

68 $$\begin{aligned} \begin{aligned}&T_{11}^{np_1}{\hat{P}}_{np_1} + T_{12}^{np_1}{\hat{Q}}_{np_1} - {\hat{P}}_{nd} = 0\\&T_{11}^{np_2}{\hat{P}}_{np_2} + T_{12}^{np_2}{\hat{Q}}_{np_2} - {\hat{P}}_{nd} = 0 \\&T_{21}^{np_1}{\hat{P}}_{np_1} + T_{22}^{np_1}{\hat{Q}}_{np_1}\; +\; T_{21}^{np_2}{\hat{P}}_{np_2}\\&\quad + T_{22}^{np_2}{\hat{Q}}_{np_2}\;- {\hat{Q}}_{nd}= 0. \end{aligned} \end{aligned}$$

These equations are valid for zero harmonic too if the transmission matrix ${\varvec{T}}_{0p}$ is taken in the form of (63).

*Boundary condition at the outlets of terminating segments* In general, the Windkessel model is employed at the outlet of a terminating vessel (Alastruey et al. 2012a; Boileau et al. 2015; Carson and Van Loon 2017), which is analogous to an electric circuit containing resistors and capacitors. In the present approach proposed, for every harmonic component with frequency $\omega _n$, the impedance load for a terminating segment *s* can be defined by single complex number $Z_{sn}^{\mathrm {out}}$ rather than by an ODE as in the traditional numerical schemes, i.e.,

69 $$\begin{aligned} P_{ns}^{(1)}(L_s) - Z_{ns}^{\mathrm {out}} Q_{ns}^{(1)}(L_s) = 0. \end{aligned}$$

For example, in the standard three-element Windkessel model, containing two resistors $R_1$ and $R_2$ and a capacitor *C*, the impedance equals $Z_{ns}^{\mathrm {out}} = R_1 + R_2/(1+{i}\omega _n R_2C)$.

The non-reflecting boundary condition can be set in a natural way for any nonzeroth harmonic component. In the proposed approach, the outlet impedance should be equal to the ratio of the pressure and flow rate in the harmonic forward propagating wave, i.e.,

70 $$\begin{aligned} Z_{ns}^{\mathrm {out}} = P^{f}_{ns}(L_s)/Q^{f}_{ns}(L_s). \end{aligned}$$

If the terminating segment is cylindrical, then the outlet impedance simply should be equal to the characteristic impedance for that segment: $Z_{ns}^{\mathrm {out}} = \tilde{Z}_{ns}=\rho c_{s}/(A_s\phi _{sn})$. If the terminating segment is approximated by a set of cones (see Sect. 4.4), then as it is seen from (50), the *P* / *Q* ratio in the forward propagating wave is $P^f/Q^f = \tilde{Z}/\left( 1-{i}\zeta \right)$. Therefore the non-reflecting outlet impedance should be

71 $$\begin{aligned} Z_{ns}^{\mathrm {out}} = \frac{\tilde{Z}_{ns}^{\mathrm {out}}}{1-{i}\zeta _{ns}^{\mathrm {out}}} \quad {\text{ where}} \quad \zeta _{ns}^{\mathrm {out}} = \frac{(a_x)_{s}^{\mathrm {out}}}{{\bar{k}}_{ns}^{\mathrm {out}}a_{s}^{\mathrm {out}}} \end{aligned}$$

Here superscript ‘out’ denotes the value at the outlet of terminating segment *s*. Expressing $P_{ns}^{(1)}(L_s)$ and $Q_{ns}^{(1)}(L_s)$ through the transmission matrix ${\varvec{T}}_{ns}$ we obtain the linear equation for ${\hat{P}}_{ns}^{(1)}$ and ${\hat{Q}}_{ns}^{(1)}$ as

72 $$\begin{aligned}&\left( (1-{i}\zeta _{ns}^{\mathrm {out}})T_{11}^s - \tilde{Z}_{ns}^{\mathrm {out}}T_{21}^s\right) {\hat{P}}_{ns}^{(1)} \nonumber \\&\quad + \left( (1-{i}\zeta _{ns}^{\mathrm {out}})T_{12}^s - \tilde{Z}_{ns}^{\mathrm {out}}T_{22}^s\right) {\hat{Q}}_{ns}^{(1)}= 0 \end{aligned}$$

Value $Z_{ns}^{\mathrm {out}}$ in condition (71) has sense of characteristic impedance of a conic vessel with constant wave velocity. The load with such impedance will absorb all forward propagating waves.

*Zeroth harmonic component* The inlet/junction/outlet conditions should be considered separately for the zeroth harmonic component. The component $P_{0s}$ for the inlet segment is set by Eq. (56). At junctions, we have the matching conditions (65). As for the outlet conditions at terminating vessel segments, the outlet resistance $R_{s}^{\mathrm {out}}$ can be set at all such segments as:

73 $$\begin{aligned} \left( {\hat{P}}_{0s} - p_{\mathrm {out}}\right) - R_{s}^{\mathrm {out}}{\hat{Q}}_{0s} = 0. \end{aligned}$$

where $p_{\mathrm {out}}$ is the pressure at which flow to the microcirculation ceases (Alastruey et al. 2012b).

Alternative way is to apply Murray’s law (Murray 1926; Sherman 1981), splitting the flow rates at a junction proportional to a power $\xi$ of lumen radius, i.e.,

74 $$\begin{aligned} {\hat{Q}}_{0d_i} = {\hat{Q}}_{0s}\frac{a_{d_i}^{\xi }}{a^{\xi }_{d_1}+\cdots +a^{\xi }_{d_{D_s}}},\qquad d_i \in {\mathcal {D}}_s. \end{aligned}$$

where $\xi = 3$. Some studies show that a value of $\xi = 3$ is optimal for laminar flow, $\xi = 2.33$ for turbulent flow and $\xi = 2.76$ is a good choice for arterial blood flow (see Olufsen 1999 and references there in). If additional information is known about splitting the flows at some junctions for particular networks, it also can be utilized instead of (74) at those junctions.

*Solution to the linearized problem* Conditions (58) [or (60)], along with conditions (65) and (72) form a closed systems of linear algebraic equations with respect to ${\hat{P}}_{ns}^{(1)}$ and ${\hat{P}}_{ns}^{(1)}$ for all $n\ne 0$. Equations (60), (65) and (73) [or (74)] form a closed system for $n=0$.

At this point, we have $N+1$ closed systems of $2N_s$ linear equations with respect to ${\hat{P}}_{ns}$ and ${\hat{Q}}_{ns}$ for $s=1,\ldots ,N_s$ and for all frequencies accounted: $\omega _n: n=0,1,\ldots ,N$. Recollect that for a negative *n* we have the same solutions but complex conjugate. Then for monitored site $x\in [0,L_s]$ of a selected vessel segment *s*, the waveform can be computed through the transmission matrix ${\varvec{T}}_s$ and the inverse Fourier transform, (34),

75 $$\begin{aligned} \begin{bmatrix} p^{(1)}_s(x,t) \\ q^{(1)}_s(x,t) \end{bmatrix} \approx \sum _{n=-N}^{N} {\varvec{T}}_{ns}(x) \begin{bmatrix} {\hat{P}}_{sn}^{(1)} \\ {\hat{Q}}_{sn}^{(1)} \end{bmatrix} {\mathrm{e}}^{{i}\omega _n t}. \end{aligned}$$

The Fast Fourier Transform (FFT) method is very useful in this case. Examples of the solutions are presented in Sect. 7.5

## Nonlinear corrections

*Nonlinear correction to solutions* Let inlet parameters ${\hat{P}}_{n}^{(1)}(0)$ and $Q_{n}^{(1)}(0)$ for the *s*th vessel segment are found for all *n* (subscript *s* is omitted in this section). The next stage is to substitute them into relation (33) in order to calculate the right-hand sides for equations at the second iteration. We should mention here that the most expensive part is to compute the $\alpha$ parameter in relation (33).

Also observe that in Fig. 1 the variation of $\alpha$ is relatively small around the value 4/3 in contrast to the larger variation of the $\gamma$ parameter. Also, note that the accuracy in the computation of the correction to the main approximation can be lower compared to the main approximation. Therefore from practical viewpoint, the easiest way is to approximate $\alpha$ by a constant value, say, 4/3. Accurate computation of the $\alpha$ parameter and study of its influence on the waveform will be addressed in the future works.

Approximating $\alpha$ by a constant we can rewrite relation (33), after omitting subscript 0 (indicating the basic state) and performing the differentiation of the product, as

76 $$\begin{aligned} {\varvec{n}}^{(2)}= \begin{bmatrix} \displaystyle -\frac{\rho }{A^{2}}\alpha \left( 2q^{(1)}q_{x}^{(1)}-(q^{(1)})^{2}\frac{A_{x}}{A}\right) -\frac{\varDelta p^{(1)}p_{x}^{(1)}}{\rho c^{2}}+\frac{4\pi \nu \gamma }{c^{2}A^{2}}\varDelta p^{(1)}q^{(1)} \\ \displaystyle -\delta _{1}\frac{A}{\rho ^{2}c^{4}}\varDelta p^{(1)}p_{t}^{(1)}\end{bmatrix}. \end{aligned}$$

Estimations show that the third addend in the first row accounting for combined correction of nonlinearity and viscosity is relatively small and can be omitted. Utilizing properties of the Fourier transform and also Eqs. (40) and (41) we can calculate the derivatives as

77 $$\begin{aligned} p_{t}^{(1)}&=\sum _{n=-\infty }^{+\infty }{i}\omega _{n}P_{n}^{(1)}{\mathrm{e}}^{{i}\omega _{n}t} \end{aligned}$$

78 $$\begin{aligned} p_{x}^{(1)}&=\sum _{n=-\infty }^{+\infty }(-{i}\omega _{n})\rho \phi _{n}^{2}\frac{Q_{n}^{(1)}}{A}{\mathrm{e}}^{{i}\omega _{n}t} \end{aligned}$$

79 $$\begin{aligned} q_{x}^{(1)}&=\sum _{n=-\infty }^{+\infty }(-{i}\omega _{n})\frac{A}{\rho c^{2}}P_{n}^{(1)}{\mathrm{e}}^{{i}\omega _{n}t} . \end{aligned}$$

After computation of ${\varvec{n}}^{(2)}(x,t)$ for the segment, we have to apply the Fourier transform (38) to relation (76) and integrate the inhomogeneous ODEs to give

80 $$\begin{aligned} \left( P_{n}^{(2)}\right) _{x}+\frac{{i}\omega _{n}\rho \phi ^{2}}{A}Q_{n}^{(2)}&=N_{n1}^{(2)} \end{aligned}$$

81 $$\begin{aligned} \left( Q_{n}^{(2)}\right) _{x}+\frac{{i}\omega _{n}A}{\rho c^{2}}P_{n}^{(2)}&=N_{n2}^{(2)} \end{aligned}$$

with zeroth initial conditions at the segment inlet: $P_{n}^{(2)}(0)=0$ and $Q_{n}^{(2)}(0)=0$. The zeroth initial condition here needs explanation. Solution to linear inhomogeneous ODEs (80) and (81) with general initial conditions can be represented as a sum of solution to homogeneous ODEs (40) and (41) with general initial conditions (which gives solution $[{\hat{P}}_{n}^{(1)}, {\hat{Q}}_{n}^{(1)}]$) and the solution to inhomogeneous ODEs with zeroth initial condition (this addend just gives solution $[P_{n}^{(2)}, Q_{n}^{(2)}]$).

ODEs (80) and (81) can be integrated numerically, for example, using Runge–Kutta methods, but we propose here a less accurate but faster method of integration based on piecewise conic approximation. Recall again that a lower accuracy is acceptable for correction to the main approximation. Solution to the inhomogeneous ODEs with zeroth initial conditions can be represented through the quadratures as

82 $$\begin{aligned}\begin{bmatrix}P^{(2)}(x) \\ Q^{(2)}(x)\end{bmatrix} &= \begin{bmatrix}T_{11}(x) \\ T_{21}(x) \end{bmatrix} \int _{0}^{x}\begin{vmatrix} N_{1}^{(2)}&T_{12} \\ N_{2}^{(2)}&T_{22} \end{vmatrix}{\mathrm {d}}x\nonumber \\&\quad + \begin{bmatrix} T_{12}(x) \\ T_{22}(x) \end{bmatrix} \int _{0}^{x}\begin{vmatrix} T_{11}&N_{1}^{(2)} \\ T_{21}&N_{2}^{(2)} \end{vmatrix}{\mathrm {d}}x . \end{aligned}$$

Subscript *n* is omitted here for brevity. To calculate entries of the ${\varvec{T}}$ matrix, we use the conic approximation described in Sect. 4.4. To calculate the quadratures, we employ the Trapezoidal rule using cones’ edge points $\{x_{0},\ldots ,x_{{N_e}}\}$ as points of discretization. Now, the flow parameters at the outlet of the *j*th element are given as

83 $$\begin{aligned} \begin{bmatrix}P^{(2)}(x_j) \\ Q^{(2)}(x_j)\end{bmatrix} \approx \begin{bmatrix}T_{11}(x_j) \\ T_{21}(x_j)\end{bmatrix}C_{1j}+ \begin{bmatrix} T_{12}(x_j) \\ T_{22}(x_j)\end{bmatrix} C_{2j} \end{aligned}$$

where

$$\begin{aligned} C_{1j}&=\sum _{i=1}^j \left( B_{1,i-1}+B_{1,i}\right) \frac{h_{i}}{2} \qquad C_{2j} =\sum _{i=1}^j \left( B_{2,i-1}+B_{2,i}\right) \frac{h_{i}}{2} \\ B_{1,i}&= \begin{vmatrix} N_{1}^{(2)}(x_i)&T_{12}(x_i) \\ N_{2}^{(2)}(x_i)&T_{22}(x_i) \end{vmatrix}\qquad \quad B_{2,i} = \begin{vmatrix} T_{11} (x_i)&N_{1}^{(2)}(x_i) \\ T_{21}(x_i)&N_{2}^{(2)}(x_i) \end{vmatrix}. \end{aligned}$$

Here ${\varvec{T}}(x_i) = {\varvec{T}}_i \,{\varvec{T}}_{i-1}\cdots {\varvec{T}}_1$, where ${\varvec{T}}_i$ is given by formula (54). In the case of $n=0$, solutions to ODEs (80) and (81) are given as

$$\begin{aligned} P_{0}^{(2)}=\int _{0}^{x}N_{01}^{(2)}{\mathrm {d}}x,\qquad Q_{0}^{(2)}=\int _{0}^{x}N_{02}^{(2)}{\mathrm {d}}x. \end{aligned}$$

Again the quadratures can be computed by the Trapezoidal rule as

84 $$\begin{aligned} \begin{bmatrix}P_0^{(2)}(x_j) \\ Q_0^{(2)}(x_j)\end{bmatrix} \approx \sum _{i=1}^{j} \begin{bmatrix}N_{01}^{(2)}(x_{j-1})+N_{01}^{(2)}(x_{j}) \\ N_{02}^{(2)}(x_{j-1})+N_{02}^{(2)}(x_{j}) \end{bmatrix} \frac{h_{i}}{2}. \end{aligned}$$

*Nonlinear corrections to the junction matching conditions* With the account for nonlinear corrections, the outlet flow parameters take the following form

85 $$\begin{aligned} P_{ns}(L_{s})&=T_{11}^{ns}{\hat{P}}_{ns}+T_{12}^{ns}{\hat{Q}}_{ns}+P_{ns}^{(2)}(L_{s}) \end{aligned}$$

86 $$\begin{aligned} Q_{ns}(L_{s})&=T_{21}^{ns}{\hat{P}}_{ns}+T_{22}^{ns}{\hat{Q}}_{ns}+Q_{ns}^{(2)}(L_{s}) \end{aligned}$$

where $P_{ns}^{(2)}$ and $Q_{ns}^{(2)}$ satisfy inhomogeneous ODEs (80)–(81) with zero initial conditions. Now, we can invoke the continuity of total pressure at a junction. Equation (85) can now be rewritten as

87 $$\begin{aligned} P_{nd}(0) = P_{ns}(L_{s})+{\mathcal {N}}_{nd},\quad d\in {\mathfrak {D}}_{s}. \end{aligned}$$

where

88 $$\begin{aligned} {\mathcal {N}}_{nd}=\frac{\rho }{2}\left( U_s^2(L_{s})\right) _{n} -\frac{\rho }{2}\left( U_d^2(0)\right) _{n} \end{aligned}$$

are nonlinear terms accounted only in the second iteration after linear values, $Q_{ns}^{(1)}$ and $Q_{ns}^{(1)}(L_{s})$, have been computed. Values $U_s^2(x)$ are computed through the inverse and direct Fourier transforms:

89 $$\begin{aligned} \left( U_s^2(x)\right) _{n}= & \frac{1}{T}\int _0^T u_s^2(x,t){\mathrm{e}}^{-{i}\omega _nt}\,{\mathrm {d}}t,\quad \nonumber \\u_s(x,t) = & \sum _{n=-\infty }^{+\infty }\frac{Q^{(1)}_{ns}(x)}{A_s(x)}{\mathrm{e}}^{{i}\omega _nt} \end{aligned}$$

where $x=0,L_s$. Here $A_{s}(0)$ and $A_{s}(L_{s})$ are, respectively, the inlet and outlet area of the *s*th segment, and $Q_{ns}^{(1)}(L_{s}) = T_{21}^{ns} {\hat{P}}_{ns}^{(1)} + T_{22}^{ns}{\hat{Q}}_{ns}^{(1)}$. Now, substituting (87), we obtain ${\mathcal {D}}+1$ linear inhomogeneous equations as

90 $$\begin{aligned} \begin{array}{rcl} T_{11}^{ns}{\hat{P}}_{ns}+T_{12}^{ns}{\hat{Q}}_{ns}-{\hat{P}}_{nd_{1}} & = & {\mathcal {N}}_{nd_1}-P_{ns}^{(2)}(L_{s}) \\ & \vdots & \\ T_{11}^{ns}{\hat{P}}_{ns}+T_{12}^{ns}{\hat{Q}}_{ns}-{\hat{P}}_{nd_{{\mathcal {D}}}} & = & {\mathcal {N}}_{nd_{{\mathcal {D}}}}-P_{ns}^{(2)}(L_{s}) \\ T_{21}^{sn}{\hat{P}}_{ns}+T_{22}^{ns}{\hat{Q}}_{ns}-{\hat{Q}}_{nd_{1}}-\cdots -{\hat{Q}}_{nd_{{\mathcal {D}}}} & = & -Q_{ns}^{(2)}(L_{s}). \end{array} \end{aligned}$$

Analogous equation can be written for a more complicated arterial junction in which more than one parent artery are connected.

*Solving the nonlinear problem* The second iteration of the network problem equations is different from the first iteration as inhomogeneous equations (90) instead of (65) are employed in the second iteration. If Murray’s law, (74), is used, then the corrected outlet flow rate also should be accounted. The total system of $2N_s$ algebraic equations with respect to ${\hat{P}}_{ns}$ and ${\hat{Q}}_{ns}$ is still linear, but most of the equations are inhomogeneous. After solving all $N+1$ systems of equations, the corrected waveforms can be computed in all necessary sites of a flow network.

## Comparison with other numerical 1D schemes for an arterial network

### Effect of accurate viscous term

First, we consider the effect of the more accurate account of viscous decay of the propagating pulse. For this purpose we compute propagation of a single Gaussian-shaped pulse along a uniform pipe having length of $10\hbox { m}$, lumen radius of $1\hbox { cm}$ and $c_0=6.17\hbox { m/}s$, considered first in Alastruey et al. (2012a) and then used for comparison of various numerical 1D schemes in Boileau et al. (2015). The flow rate at the inlet is set as $q^{\mathrm {in}}(t) = 1\times \exp \{-(t-0.05)^2/0.01^2\} \hbox { cm}^3$/s. Non-reflection boundary condition is set at the outlet. The standard blood properties are used, i.e., $\rho = 1.04\hbox { g}/\hbox {cm}^3$ and $\nu = 0.04 \hbox { cm}^2$/s. The velocity profile at inlet is taken as $u(r,x,t) = {\bar{u}}(x,t)(\zeta +2)/\zeta \,\left[ 1 - (r/a)^{\zeta } \right]$ Alastruey et al. (2012a), Boileau et al. (2015) with $\zeta = 9$ that corresponds to $\gamma = 11$.

**Fig. 4:**
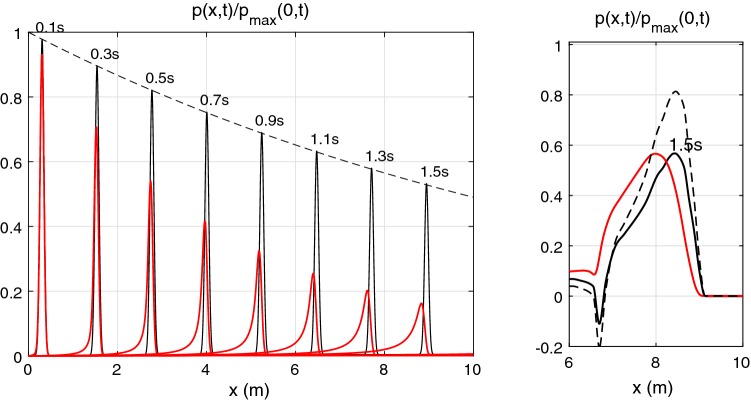
Left: propagation of a Gaussian-shape pulse along a 10 m pipe of the 2 cm diameter with $c_0 = 6.17\,\hbox {m/s}$. Black: $\gamma = 11$; Red: Womersley’s profile-based decay. Dashed line indicates the theoretical decay (93). Right: propagation of a realistic waveform along the same pipe at $t=1.5\,\hbox {s}$ for $\gamma =11$ (black solid), for $\gamma =4$ (black dashed), and for Womersley’s profile-based decay (red)

The velocity reaches at a peak value of 1 cm/s which is much smaller than the wave velocity. Therefore nonlinear effects are small and we can apply the linearized approach described in Sects. 4 and 5. In our approach, we have to deal with periodic pulses. Thus, we select a period $T = 4\hbox { s}$ that is significantly greater than the time of pulse propagation along the pipe, $t = 10/6.17 = 1.62\hbox { s}$. The results of computation with the frequency independent $\gamma = 11$ are plotted using black lines in Fig. 4 (left). One can compare these results with that of Alastruey et al. (2012a), Boileau et al. (2015) and confirm that pulse peak is identical to the published results.

Using the approach developed here, this decay can be evaluated analytically. The wave number that accounts for viscosity is

91 $$\begin{aligned} k = \frac{\omega \phi }{c_0} = \frac{\omega }{c_0}\sqrt{1-{i}\frac{\omega _{\nu }}{\omega }},\quad {\text{ where}} \quad \omega _{\nu } = \frac{2\pi \gamma \nu }{A_0}. \end{aligned}$$

For the case under consideration $\omega _{\nu } = 2\pi \times 0.14 = 0.88\hbox { s}^{-1}$ that is small in comparison with frequency of all harmonic components except the zeroth one. Taking into account the fact that $\omega _{\nu }$ is small and expanding $\phi$ gives

92 $$\begin{aligned} k \approx \frac{\omega }{c_0}\left( 1-\frac{{i}}{2}\frac{\omega _{\nu }}{\omega }\right) = \frac{\omega }{c_0}-{i}\frac{\omega _{\nu }}{2\,c_0} = \frac{\omega }{c_0}-{i}\frac{\pi \gamma \nu }{c_0A_0} \end{aligned}$$

i.e. all harmonic components with frequency much higher than $\omega _{\nu }$ are decaying at the same rate as

93 $$\begin{aligned} {\mathrm{e}}^{{i}\omega t - {i}kx} \approx {\mathrm{e}}^{{i}\omega (t - x/c_0) -\textstyle \frac{\pi \gamma \nu }{c_0A_0}\scriptstyle x }. \end{aligned}$$

Thus, if such harmonic components mainly constitute the pulse, then the pulse will propagate, preserving its shape and decaying exponentially with the coefficient $\frac{\pi \gamma \nu }{c_0A_0}$. This coefficient is equal to $0.0713\hbox { m}^{-1}$ for the problem considered here. The narrow pulse considered here is rich in high-frequency harmonics for which decay given by (93) is accurate. The analytical exponential decay of the pulse peak is plotted by a dashed line in Fig. 4.

When correctly accounted for viscous term, i.e. when the $\gamma$ parameter is calculated accurately based on Womersley’s solution (see Flores et al. 2016 and Sect. A), change in shape and amplitude of the propagating pulse as shown in Fig. 4 (left) is observed. One can see here that the pulse is decaying noticeably faster, its shape is modified with formation of pulse tail, and the pulse peak propagates slightly slower than in the case of constant $\gamma$. Such big difference in pulse decay is obtained as we have considered a very narrow pulse instead of a realistic waveform, observed in arteries. This narrow pulse is rich in high-frequency harmonics with $\gamma$ value greater than 11. In a realistic waveform, first five harmonic components contain 95% of the pulse energy, whereas in the narrow Gaussian pulse used in Alastruey et al. (2012a), Boileau et al. (2015), the number of harmonic components containing 95% of the pulse energy is 32. For a realistic waveform computed with the constant gamma $\gamma = 11$ and based on Womersley profiles give approximately the same decay of the pulse peak as seen in Fig. 4 (right). Nevertheless there is some difference in the pulse shape and propagation speed between the constant $\gamma$ approximation and Womersley’s profile-based treatment of the viscous term. A large difference is observed in the narrow negative peak in Fig. 4 (right) as it is formed by higher frequencies.

For the sake of comparison, a waveform propagation computed with the $\gamma =4$ (the value often taken in 1D arterial network modelling) is plotted using the dashed line in Fig. 4 (right). One can see that $\gamma =4$ underestimates the pulse peak decay in the pipe with $a_0=1\hbox { cm}$. This indicates that in a large artery like aorta $\gamma = 11$ is a reasonably effective viscous parameter, averaged over accurate $\gamma$ values of the main harmonics constituting a typical waveform. At the same time, in a narrower vessel, the effective constant $\gamma$ should be smaller than 11 as the Womersley’s profile-based $\gamma$ values for the main harmonics are smaller (see Fig. 13).

### Nonlinear effects in a uniform pipe

*Comparison with the analytical solution* First, we estimate the accuracy of the numerical integration (84) against the analytical solution for a uniform pipe and inviscid flow. Equation (32) may be written in the following form:

94 $$\begin{aligned} \begin{array}{ll} {\hat{p}}_{x}^{(2)}+c_0^{-1}{\hat{q}}_{t}^{(2)} = -2\alpha {\hat{q}}^{(1)}{\hat{q}}^{(1)}_{x}- {\hat{p}}^{(1)}{\hat{p}}^{(1)}_{x}, & \varDelta p = \displaystyle \rho c_0^2\,{\hat{p}}, \\ {\hat{q}}_{x}^{(2)}+ c_0^{-1}{\hat{p}}_{t}^{(2)} = -\delta _{1} c_0^{-1}{\hat{p}}^{(1)}{\hat{p}}^{(1)}_{t}, &\quad q = \displaystyle {A_0}{c_0}\,{\hat{q}}. \end{array} \end{aligned}$$

Here the pressure and flow rate are expressed through dimensionless variables ${\hat{p}}$, ${\hat{q}}$. Equation (94) admits an analytical solution, for example, for the following linear wave

95 $$\begin{aligned} {\hat{p}}^{(1)} = {\hat{q}}^{(1)} = \varepsilon \displaystyle \left( \xi +(1-\xi )\cos (\omega t-kx)\right) ,\qquad k={\omega }/{c_0} \end{aligned}$$

where $\varepsilon$ is an dimensionless amplitude factor: $\varepsilon = \varDelta p^{(1)}_{\max }/\rho c_0^2$; parameter $\xi$ determines the ratio of the constant and oscillating parts. Solutions to PDEs (94) satisfying boundary condition ${\hat{p}}^{(2)}(0,t)={\hat{q}}^{(2)}(0,t)=0$ are:

96 $$\begin{aligned} {\hat{p}}^{(2)}&= B_1^+\sin (\omega t - kx)(kx) + B_1^- \sin (\omega t)\sin (kx)\nonumber \\&+ B_2^+\sin (2\omega t - 2kx)(2kx) + B_2^- \sin (2\omega t)\sin (2kx)\nonumber \\ {\hat{q}}^{(2)}&= B_1^+\sin (\omega t - kx)(kx) - B_1^- \sin (\omega t)\sin (kx)\nonumber \\&+ B_2^+\sin (2\omega t - 2kx)(2kx) - B_2^- \sin (2\omega t)\sin (2kx) \end{aligned}$$

where

97 $$\begin{aligned} B_1^{\pm } = -\varepsilon ^2\xi (1-\xi )\frac{2\alpha +1\mp \delta _1}{2}, \qquad B_2^{\pm } = -\varepsilon ^2\xi ^2\frac{2\alpha +1\mp \delta _1}{8}. \end{aligned}$$

**Fig. 5:**
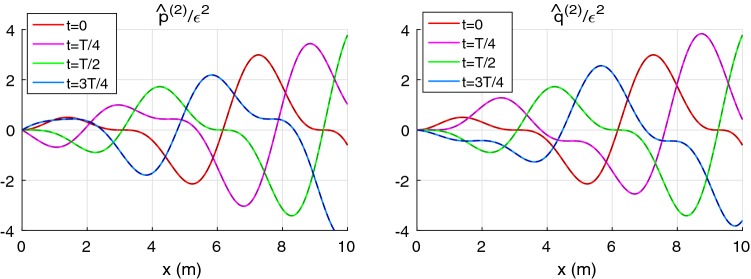
Dimensionless nonlinear corrections for the pressure ${\hat{p}}^{(2)}/\varepsilon ^2$ (left) and the flowrate ${\hat{q}}^{(2)}/\varepsilon ^2$ (right) for the waveform, (95), with $\xi =0.5$. Solid lines: the exact solution, (96) and (97); dashed darker lines: results of the numerical calculations, (84) with $h=2\hbox { cm}$

The dimensionless nonlinear correction parameters along the 10-m pipe problem (see previous subsection) for the linear waves described by Eq. (95) are presented in Fig. 5. One can see that exact and numerically obtained curves are indistinguishable. Analysis shows that relative error of the numerical computation is 0.06% for an element length of $h=2\hbox { cm}$.

Solution (96)–(97) helps us to compare contribution of different nonlinear parameters in a waveform. The growing part of the solution contains the following factor

98 $$\begin{aligned} B^+_{1,2} \propto 2\alpha + 1 - \delta _1. \end{aligned}$$

The convection term, $(\alpha q^2/A)_x$, mainly contributes to $B^+_{1,2}$ and $2\alpha = 8/3$ for the Poiseuille profile and the term $\varDelta A/A_0 p_x$ in Eq. (27) is unity in relation (98). The value of $\delta _1$ in the constant stiffness constitutive law is 0.5. Thus, contribution of nonlinearity, related to the constitutive law, is the small in this case. Nevertheless the constitutive law (20) can give large negative value of $\delta _1$. In such situation, contribution of the vessel wall to the nonlinear corrections can be much higher and it can additionally increase the growth of nonlinear corrections.

### Accuracy for a tapering vessel

**Fig. 6:**
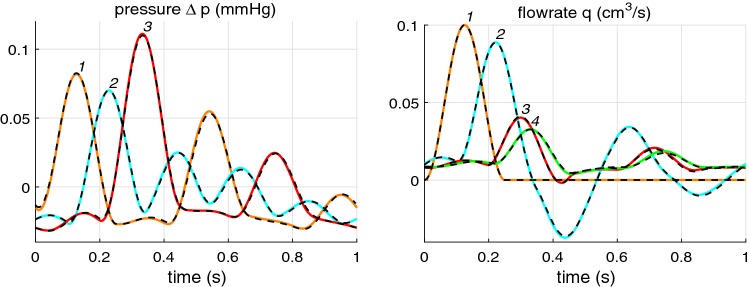
Pressure (left) and flow rate (right) waveforms computed at the inlet, *1*, midpoint, *2*, outlet, *3*, of the first pipe and outlet of the conic segment, *4*. The pressure waveform in cite *4* not presented as it is almost identical to that of at site *3*. Coloured curves are computed using the method described in Carson and Van Loon (2017), dashed black curves are computed using the proposed method

Here, we consider the simplest network which comprises a 1-m-long pipe with uniform cross section, connected to a tapering segment and the outlet of the tapering part is connected to another 1-m-long pipe. Such long inlet pipe is chosen to have forward and reflected pulses clearly separated in time (see Fig. 6). The dimensions of the tapering segment correspond to the Right Carotid segment used in the arterial network described in Mynard and Nithiarasu (2008) and used in Sect. 4.4. Its inlet and outlet diameters, respectively, are 6.75 mm and 3.5 mm, and its length is 9.4 cm. The shape of the tapering segment here is approximated using a truncated cone. The diameters of the first and third pipe are equal, respectively, to the inlet and outlet diameters of the tapering segment. The dependance of PWV $c_0$ on lumen diameter is taken from Blanco et al. (2015). The non-reflecting boundary condition (69)–(70) is set at the outlet of the network. A flow rate with a bell-like shape is set at the inlet of the network, i.e.,

99 $$\begin{aligned} q^{\mathrm {in}}(t) = \left\{ \begin{array}{ll} q_{\max }\frac{1}{2} \left( 1 + \cos \left[ (2\pi /\varDelta )(t-\frac{1}{2} \varDelta )\right] \right) & 0\le t<\varDelta \\ 0 & \varDelta \le t\le T \\ \end{array}\right. \end{aligned}$$

where the period, $T=1\hbox { s}$, and the pulse duration, $\varDelta = T/4 = 0.25\hbox { s}$.

The pressure and flow rate waveforms are computed at four sites: *1*—the inlet, *2*—midpoint and *3*—outlet of the first pipe and *4*—the outlet of the tapering segment. The waveforms computed by the numerical scheme described in Carson and Van Loon (2017) are shown in Fig. 6 using coloured lines and waveforms computed by the proposed method are shown using dashed black lines. The inlet flow amplitude is taken sufficiently small with $q_{\max }=0.1\hbox { cm}^3$/s to ensure that the process is linear and $\gamma =4$ is employed in both computations. One can see that agreement between proposed method and numerical computations is excellent and this proves the accuracy of the proposed method, at least for the linear process.

**Fig. 7:**
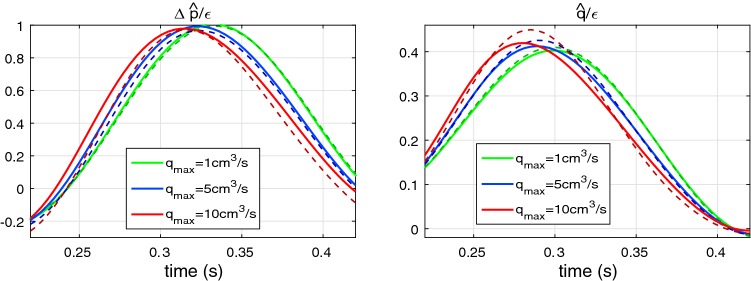
Part of the normalized pressure (left) and flow rate (right) waveforms computed at site *3* (inlet of the tapering segment) in the vicinity of the main peak for different peak flow rate values (indicated in the legend). The solid coloured lines are computed using the method in Carson and Van Loon (2017), and the dashed lines are computed using the proposed method

We now discuss how the waveforms depend on the inlet peak flow amplitude, $q_{\max }$. A part of the waveforms in site *3* (inlet of the tapering segment), in the vicinity of the main peak are shown in Fig. 7. They are shown in a normalized form with $\varDelta {\hat{p}} = p/\rho c_0^2$ and ${\hat{q}} = q/(A_0c_0)$, which are proportional to the amplitude parameter, $\varepsilon = q_{\max }/(A_0c_0)$. Therefore, dividing by $\varepsilon$ we obtain normalized waveforms having approximately the same amplitude distorted only by the nonlinear factors. One can see that the pressure peaks are shifted forward with the increase in amplitudes. The waveforms computed by the proposed method behaves similarly to that of the numerical computation, but their shift is smaller when the amplitude approaches $q_{\max }=10\hbox { cm}^3$/s.

**Fig. 8:**
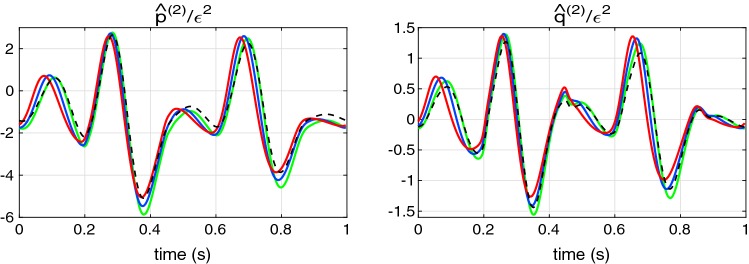
Normalized nonlinear corrections for the pressure (left) and flow rate (right) waveforms computed at site *3* for different flow rate peak values of the inlet flow. The colours are the same as in Fig. 7. Normalized waveforms ${\hat{p}}^{(2)}/\varepsilon ^2$ and ${\hat{q}}^{(2)}/\varepsilon ^2$ computed using the proposed method is independent of the amplitude and indicated by black dashed curve

In Fig. 8 one can see the evaluation of nonlinear corrections $p^{(2)}$ and $q^{(2)}$ with the growth of the inlet flow amplitude. As the second-order correction are proportional to the square of the amplitude, it is natural to plot them in the normalized form ${\hat{p}}^{(2)}/\varepsilon ^2$, ${\hat{q}}^{(2)}/\varepsilon ^2$. In the proposed method, if we account only the second-order corrections in equation (32), the normalized waveform is amplitude independent. They are indicated by the black dashed line in Fig. 8. To compute the nonlinear corrections using standard 1D methods, in particular, using the method in Carson and Van Loon (2017), we carry out the following procedure. First, we compute the waveform for an extremely low amplitude. We take $q_{\max ,\mathrm {ref}} =10^{-3}\hbox { cm}^3$/s, compute waveforms $p_{\mathrm {ref}}$ and $q_{\mathrm {ref}}$ and use it as a reference linear solution. Then, we calculate the waveforms *p* and *q* at a realistic amplitude $q_{\max }$ and calculate the nonlinear correction by the formula,

100 $$\begin{aligned} p^{(2)}= & \varDelta p - \varDelta p_{\mathrm {ref}}\,(q_{\max }/q_{\max ,\mathrm {ref}}),\nonumber \\ q^{(2)}= & q-q_{\mathrm {ref}}\,(q_{\max }/q_{\max ,\mathrm {ref}}). \end{aligned}$$

Here $\varDelta p = p\, -\, {\bar{p}}$, where ${\bar{p}} = (1/T)\int _0^T p\,{\mathrm {d}}t$ is the mean pressure. The results of the computation are plotted in Fig. 8 in the normalized form (solid lines). The distortion of this corrections with the growth of amplitude indicates influence of the higher-order corrections in Eq. (32).

Note that the nonlinear distortion of the pulse is high in this network due to a long ($L=1\hbox { m}$) pipe connected to a tapering segment. The consideration based on Riemann equation, describing formation of a shock wave confirms this. The nonlinear distortion is accumulated during its propagation along a long uniform pipe as the higher harmonics generated by the nonlinear effect propagate with the same speed along a uniform pipe. Any change in geometry like tapering, branching, PWV variation, etc. will cause discrepancy between the phase velocity of the different harmonics that will prevent accumulation of nonlinear distortion. Therefore, in the real arterial system such distortion should be smaller.

### Comparison with experimental results

**Fig. 9:**
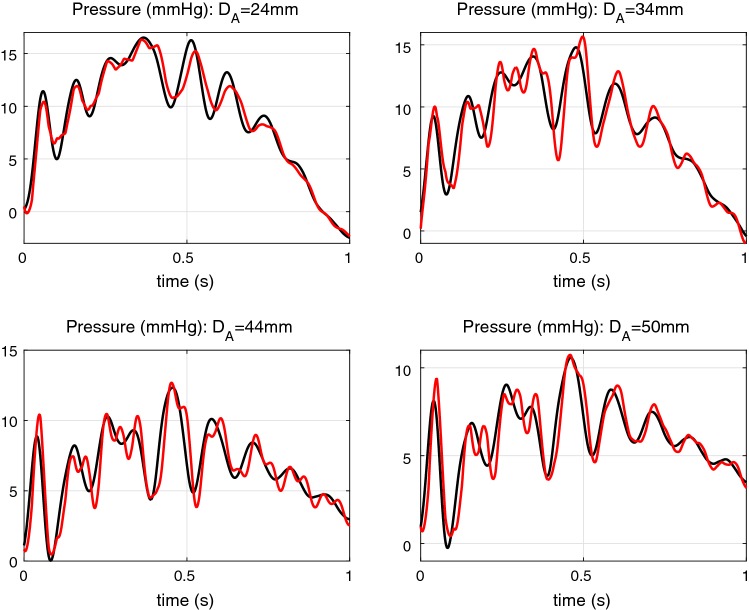
Comparison of measured (black) and computed (red) pressure waveforms. The experimental setup is described in Sazonov et al. (2017)

In the work Sazonov et al. (2017), an in vitro setup is described for studying pulse reflection from synthetic aneurysms of different diameters. Details of the setup, parameters, inlet flow rate waveforms and measured pressure waveforms are presented in Sazonov et al. (2017). A comparison of the measured pressure waveforms and waveforms computed by the proposed method is depicted in Fig. 9 by black and red lines, respectively. The waveforms are compared for the site $25\hbox { cm}$ from the inlet of the main pipe, i.e. at the midpoint of the 50-cm-long pipe preceding the segment with an aneurysm. The comparison is carried out for all aneurysm diameters, $D_A$, considered in Sazonov et al. (2017), i.e., 24 mm, 34 mm, 44 mm and 50 mm.

All the pipes in the experimental setup, except one with the aneurysm have a constant diameter. Therefore, the calculation of the transmission matrix ${\varvec{T}}$ is straightforward using (45). The element size for nonlinear corrections is taken equal to 2 cm. For a 14 cm segment with an aneurysm, the element size is taken equal to 0.5 cm, both for the linear problem and for nonlinear corrections. The viscosity factor $\gamma$ is taken frequency dependent and is computed by the approximation, (130). The nonlinearity coefficients are: $\alpha = 4/3$, $\delta _1 = 1/2$. In the experiments described in Sazonov et al. (2017), single pulses are used because of the strong reflection from the outlet of the pipe network. As in the proposed method we have to deal with periodic signals, the period is taken large (4 s), and non-reflecting boundary condition is set at the outlet of the pipe network. The small discrepancy between computed and measured waveforms in Fig. 9 is caused by the uncertainty of mechanical parameters of some elements of the setup and uncertainty of the inlet boundary conditions.

### Application of the method to arterial networks

**Fig. 10:**
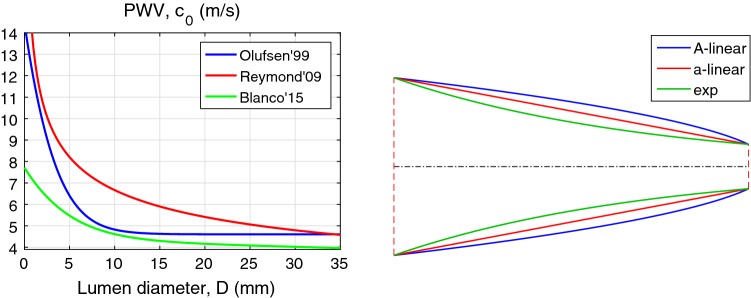
Left: The $c_0$ vs lumen diameter *D* approximations. Blue: Olufsen (1999), Mynard et al. (2010), Mynard and Smolich (2015); Red: Reymond et al. (2009); Green: Blanco et al. (2015). Right: Different types of vessel shape approximation. Blue: *A*(*x*) is linear. Red: *a*(*x*) is linear. Green: *A*, *a* are exponential

In most arterial network models, the exact variation of the blood vessel parameters is not specified as subject-specific information is very difficult to obtain (see for eg. Mynard and Nithiarasu 2008). In many cases, we have to interpolate the vessel parameter variations along the length of the vessel. For example, the lumen area *A* and the wave speed *c* [or the $\beta$ parameter in relation (9)] require interpolation along the length. Often *A*(*x*) is linearly interpolated along the vessel length (Mynard and Nithiarasu 2008) and the same approach is followed for $\beta$ parameter if its inlet and outlet values are given. In some works, the authors approximate only *A*(*x*) and apply one of the known approximations for *c*(*a*) (recall $a = \sqrt{A/\pi }$ is the lumen radius). Such approximations are mainly employed in 1D modelling, which can be written in the following forms:

101 $$\begin{aligned} c^2 = b_1{\mathrm{e}}^{-\lambda a}+b_2&\quad {\text{ with}}\,\, b_1=188.7, b_2=21.19, \lambda =9 \end{aligned}$$

102 $$\begin{aligned} c^2 = b'_1{\mathrm{e}}^{-\lambda _1a}+b'_2{\mathrm{e}}^{-\lambda _2a}&\quad {\text{ with}}\,\, b'_1=40.41, b'_2=19.10, \lambda _1=5.053, \lambda _2=0.1114 \end{aligned}$$

103 $$\begin{aligned} c = b^{\prime \prime }_1/(20a)^{b^{\prime \prime }_2}&\quad {\text{ with}}\,\, b^{\prime \prime }_1=13.3, b^{\prime \prime }_2=0.3 \end{aligned}$$

where the lumen radius *a* should be in centimetres, and the obtained PWV *c* is in m/s. Approximation (101) is proposed in Olufsen (1999) and its parameters for systemic arteries are listed in Mynard and Smolich (2015). Approximation (102) is proposed in Blanco et al. (2015) and approximation (103) is proposed in Reymond et al. (2009). Here, 20*a* equals to the lumen diameter *D* expressed in mm. These approximations for *c*(*a*) are plotted in Fig. 10(left).

Different types of lumen radius variation, adapted by different authors, are depicted in Fig. 10(right). They are: the area is linearly interpolated as in Mynard and Nithiarasu (2008) (blue), the radius is linearly interpolated as shown in red and exponential interpolation of area is shown in green. One can see that the linear interpolation of the area produces a convex and non-realistic shape. Thus, other types of interpolation of *A*(*x*) are better approximations. Nevertheless, for a typical artery, the difference between inlet and outlet radii is much smaller and discrepancies between the interpolations are hardly important.

**Fig. 11:**
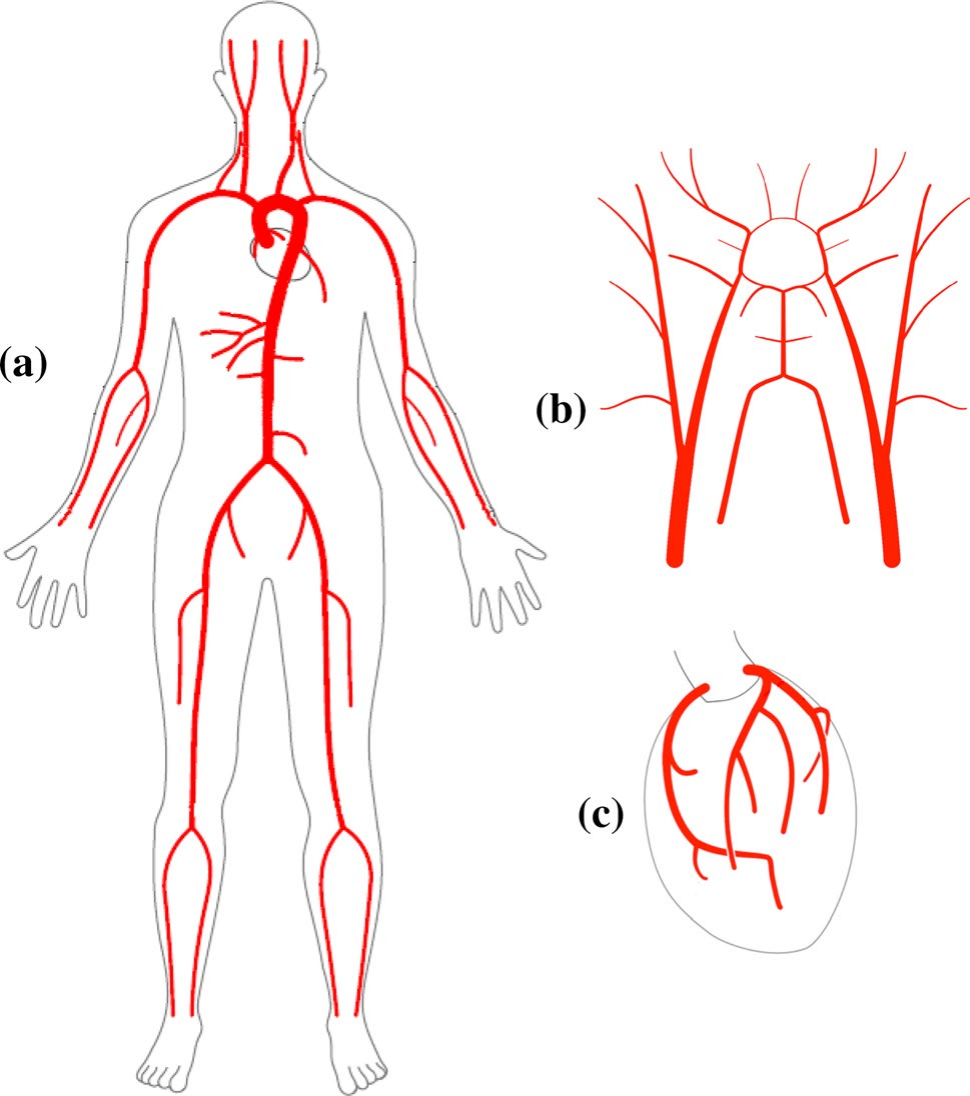
Arterial network model described in Mynard and Smolich (2015) and used here for simulation: main arteries (**a**), cerebral arteries (**b**), coronary arteries (**c**)

For assessing the accuracy of the proposed method, we compare our results against the numerical modelling results of the human arterial network using a well established method, described in Mynard and Nithiarasu (2008). Here we use the arterial network described in Mynard and Smolich (2015), which contains 107 blood vessel segments as shown in Fig. 11. The dependence of the PWV on lumen radius is calculated using Eq. (102). The flow rate $q^{\mathrm {in}}(t)$ is imposed at the inlet of the first segment as in Blanco et al. (2015); Boileau et al. (2015) with the heartbeat period of $T = 1\hbox { s}$. All the tapering segments are approximated by truncated cones. We have set constant values $\gamma = 4$ and $\alpha =1$ as in the computational model Mynard and Nithiarasu (2008). The constitutive relation is taken in the form of relation (9) and thus the nonlinearity parameter $\delta _1=0.5$. The three-element Windkessel (lumped) model is employed as terminal boundary conditions.

**Fig. 12:**
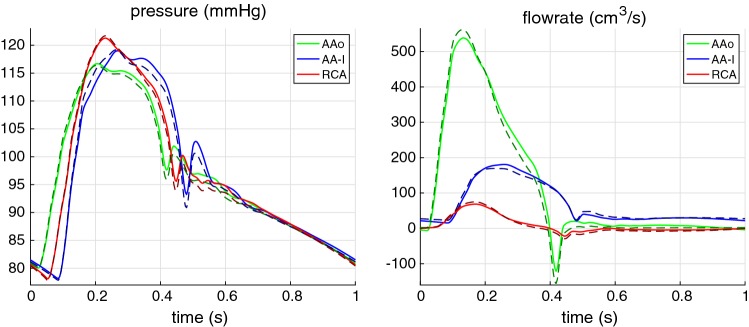
Pressure wave form (left) and flow rate waveform (right). Computed waveforms at the beginning of aortic arc (green), at the beginning of abdominal aorta (blue) and at the midpoint of the right carotid (red). Waveforms computed by the model in Mynard and Nithiarasu (2008) are shown using solid lines and those computed by the proposed method are shown by dashed lines

**Fig. 13:**
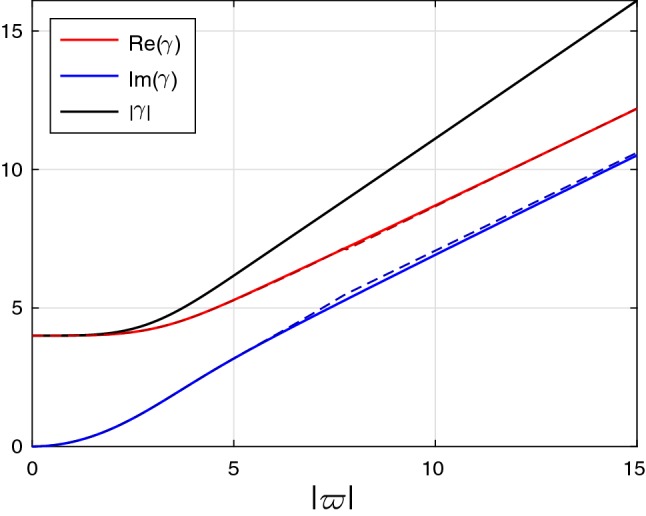
Dependence of real part (red), imaginary part (blue) and absolute value (black) of the $\gamma$ parameter on the Womersley number $|\varpi |= a_0\sqrt{\omega /2\nu }$. Dashed lines indicate an approximation (131)

The comparison of results between the present and numerical computations is shown in Fig. 12. Observe that the proposed method gives results very close to that computed by the method used in Mynard and Nithiarasu (2008). There are some small discrepancies near the peak and in the decaying part of the pulse wave, which can be attributed to the computational errors. The numerical model of Mynard and Nithiarasu (2008) needs about 300 s to compute the first 3 heartbeat cycles. The proposed method needs only 6 s to compute the correct waveforms, including the nonlinear corrections. The model proposed in Carson and Van Loon (2017) needs 50 s to compute waveforms in the same networks. Overall, the proposed method is fast and as accurate as the numerical models mentioned.

## Discussion and conclusions

Many of the currently used numerical models for arterial flow do not account correctly for the viscous friction, especially for a pulsating flow. It is obvious that several different values of viscosity coefficient $\gamma$ are employed by currently used models. The nonlinear convection term used in the mathematical models in the past is not rigorous for a pulsating flow. In addition, a variety of constitutive laws are employed for vessel walls, some of which resulted in nonlinear terms in the governing equations. All these variations in mathematical model suggest that there is no consensus among researchers on how to deal with nonlinear effects when solving for flow in arterial networks. Since the nonlinearity is often small, all the treatments used in the literature appear to work without too much disagreement in certain flow regimes (low amplitude waves). However, we have proposed a generalization of the nonlinear terms through corrections that may be applicable for all flow regimes.

A novel method, based on the perturbation technique and fast Fourier transform (FFT) in solving the 1D blood flow equations, has been proposed in the present work. The proposed method makes the FFT competitive against traditional space–time numerical schemes in terms of both robustness and speed. In contrast to the FFT approach described in Flores et al. (2016), the proposed method can be applied to an arbitrary arterial network, containing tapering vessels, vessels with stenosis and aneurysms, and to a high amplitude waveform in which the nonlinear effects are relevant. As demonstrated by the results, the proposed method is faster than competing methods and it is accurate. It accounts for viscous effects more accurately than any existing space–time methods and more importantly the viscous coefficient, $\gamma$, is automatically calculated for different flows and physical conditions. The proposed method simplifies boundary conditions required at the terminal vessels. Thus, we believe that this method can be an alternative and potentially a more effective tool for 1D modelling of blood flow in arterial networks.

Although the proposed method is a substantial improvement to the existing methods, it requires further development in the following area. Further attention is required to deal with viscoelastic effects, blood mass loss due to smaller branches, porous nature of arteries and application to clinical environment.

## Appendix 1: Womersley type solution in a uniform cylindrical pipe with flexible walls

Consider a small amplitude harmonic wave in a flexible cylindrical pipe. Introduce cylindrical coordinates: *x* is the axial direction, *r* is the radial direction. The Navier–Stokes and continuity equations for radially symmetric flow can be written in the form:

104 $$\begin{aligned} u_{t}+uu_{x}+vu_{r}+\rho ^{-1} p_{x}&=\nu \left[ u_{xx}+{r}^{-1} (ru_{r})_{r}\right] \end{aligned}$$

105 $$\begin{aligned} v_{t}+uv_{x}+vv_{r}+\rho ^{-1}p_{r}&=\nu \left[ v_{xx}+\left( {r}^{-1}(rv)_{r}\right) _{r}\right] \end{aligned}$$

106 $$\begin{aligned} u_{x}+{r}^{-1}(rv)_{r}&=0 \end{aligned}$$

where *u*(*r*, *x*, *t*) is the axial velocity, *v*(*r*, *x*, *t*) is the radial velocity, *p*(*r*, *x*, *t*) is the pressure. Subscripts *t*, *x*, *r* denote partial derivative with respect to *t*, *x*, *r*, respectively. Boundary conditions at the pipe wall $r=a(x,t)$ are:

107 $$\begin{aligned} u(a) =0,\qquad v(a) =a_{t}. \end{aligned}$$

In a rigid pipe ($a\equiv \mathrm{const}$), this boundary value problem admits exact solution for a harmonic perturbation: $[u,v,p]^{\mathrm{T}}=[{\hat{u}}(r),{\hat{v}}(r),{\hat{p}}(r)]^{\mathrm{T}}\exp \left\{ {i}\omega t\right\}$ Womersley (1955), i.e.,

108 $$\begin{aligned} {\hat{u}}=\frac{{\hat{p}}_{x}}{{i}\omega \rho }\left( 1-\frac{J_{0}(\varkappa r)}{J_{0}(\varkappa a)}\right) , \qquad {\hat{v}} \equiv 0,\qquad {\hat{p}}_x \equiv \mathrm{const} \end{aligned}$$

where ${\hat{p}}_x$ is the amplitude of the pressure gradient; $J_0$ is the Bessel function of order 0 and

109 $$\begin{aligned} \varkappa =\sqrt{\frac{-{i}\omega }{\nu }}=\sqrt{\frac{\omega }{\nu }}{\mathrm{e}}^{-{i}\pi /4} \end{aligned}$$

is a complex viscous shear wave number. Any non-harmonic perturbation can be represented as a sum of harmonic perturbations. Note that solution (108) is not a wave like solution: the velocity is independent of *x*, i.e. the perturbation does not propagate along the pipe but appears in the same phase in the full pipe as soon as the pressure gradient is applied.

If the pipe is not rigid, then a harmonic wave can propagate along the length, i.e.,

110 $$\begin{aligned}{[u(x,r,t),v(x,r,t)]}^{\mathrm{T}} = [u(r),v(r)]^{\mathrm{T}} {\mathrm{e}}^{{i}\omega t - {i}k x} \end{aligned}$$

where $k=\omega /c$ is the wave number, *c* is the wave speed. Due to viscous losses, the wave is exponentially decaying; this decay is described by the imaginary part of *k* as

111 $$\begin{aligned} {\mathrm{e}}^{{i}\omega t - {i}k x} = {\mathrm{e}}^{ {i}\omega t-\mathrm {i}\mathrm {\,Re\,}k\,x} {\mathrm{e}}^{ {\mathrm {Im}} k\,x} \end{aligned}$$

where ${\mathrm {Im}} k<0$. The pressure gradient is not constant anymore, but it varies harmonically in time and space, i.e., $p_{x}={i}k\varDelta p(r)\exp \left\{ {i}\omega t - {i}k x\right\}$ where $\varDelta p = p - p_0$.

In an elastic pipe, the boundary value problem does not admit an exact solution; hence, we have to search for an approximate solution in the case of small wave amplitude and narrow pipe compared to the wavelength. Thus we introduce two small parameters:

$$\begin{aligned} \epsilon =\frac{ u_{\max } }{c_0},\qquad \delta =k a_0 = \frac{\omega }{c_0} a_0. \end{aligned}$$

The first one is the Mach number—amplitude parameter and the second one is proportional to the axial to radial velocity ratio. Here again subscript 0 refers to the basic, stress free state for $a_0,A_0$ and to the wave velocity of small perturbation for $c_0$. We now replace *c* and $c_{0}$, respectively by $c/\delta$ and $c_{0}/\delta$. This is equivalent to that of replacing *k* with $\delta k$. We now represent the solution as a sum of Womersley solution and a small perturbation to it. We refer the variables of the Womersley solution with hat and the perturbation variables we refer with a tilde. Thus we search the solution in the following form:

112 $$\begin{aligned} u&=\epsilon \left( {\hat{u}}(r)+\delta \tilde{u}(r)\right) {\mathrm{e}}^{{i}\omega t-\mathrm {i}\delta kx} \end{aligned}$$

113 $$\begin{aligned} v&=\epsilon \delta \, \tilde{v}(r)\,{\mathrm{e}}^{{i}\omega t-\mathrm {i}\delta kx} \end{aligned}$$

114 $$\begin{aligned} \varDelta p&=\frac{\epsilon }{\delta } \rho c_0 \left( {\hat{\psi }}+\delta ^2 {\tilde{\psi }}(r)\right) {\mathrm{e}}^{{i}\omega t-\mathrm {i}\delta kx} \end{aligned}$$

115 $$\begin{aligned} \varDelta a&= \epsilon \delta \,\tilde{a}\,{\mathrm{e}}^{{i}\omega t-\mathrm {i}\delta kx},\quad \varDelta a = a - a_0 \end{aligned}$$

where functions ${\hat{\psi }}$ and ${\tilde{\psi }}$ having dimension of velocity are introduced to simplify comparison of different terms in equations. In this solution, all the variables have the amplitude factor $\epsilon$ and the perturbation terms additionally have a factor $\delta$. Representation of the pressure has a more complicated structure, necessary to balance the different order terms in the equations as it is shown below. Substituting solutions (112)–(114) into Eqs. (104)–(106) and keeping only the leading terms and omitting temporally the oscillation factor, we obtain

116 $$\begin{aligned} \epsilon {i}\omega {\hat{u}} - \epsilon c_{0}{i}k {\hat{\psi }}&= \epsilon \nu {r}^{-1} (r {\hat{u}}_r)_{r} + \mathrm {H.O.T.} \end{aligned}$$

117 $$\begin{aligned} \epsilon \delta {i}\omega \tilde{v}+ \epsilon \delta c_{0} {\tilde{\psi }}_r&=\epsilon \delta \nu \left( {r}^{-1}(r\tilde{v})_{r}\right) _{r} + \mathrm {H.O.T.} \end{aligned}$$

118 $$\begin{aligned} - \epsilon \delta {i}k{\hat{u}} + \epsilon \delta {r}^{-1}(r \tilde{v})_{r}&= \mathrm {H.O.T.} \end{aligned}$$

where H.O.T. means the higher-order terms with respect to small parameters $\delta$ and $\epsilon$.

In Eq. (116), the leading terms are of the order of $\epsilon$, whereas in (117) and (118) they are of the order of $\epsilon \delta$. The leading terms in Eq. (116) coincide with the equation for the rigid pipe. Introducing the pressure amplitude of the wave as $\varDelta {\hat{p}} = \rho c_0 {\hat{\psi }}$, we obtain a solution similar to (108) for the amplitude of axial velocity. After integration for the flow rate, we have

119 $$\begin{aligned} {\hat{u}}=\frac{\varDelta {\hat{p}}}{\rho c_0 }\left( 1-\frac{J_{0}(\varkappa r)}{J_{0}(\varkappa a_0)}\right) , \quad {\hat{q}}=\frac{\varDelta {\hat{p}}}{\rho c_0 }A_0\left( 1-2\frac{J_{1}(\varkappa a_0)}{\varkappa a_0 J_{0}(\varkappa a_0)}\right) . \end{aligned}$$

We can show that corrections to these values have an order $\delta ^2$ only.

Substituting solution (119) into Eq. (118), we can calculate the radial velocity as (if the wall is not rigid):

120 $$\begin{aligned} \tilde{v}(r)=-{i}k \frac{\varDelta {\hat{p}}}{\rho c_0 }\left[ \frac{r}{2}-\frac{ J_{1}(\varkappa r)}{\varkappa J_{0}(\varkappa a_{0})}\right] . \end{aligned}$$

Correction to the pressure $\tilde{p} = \rho c_0 {\tilde{\psi }}$ can be calculated after substitution of solutions (120) into Eq. (117) and solving with respect to $\psi$. This will give pressure variation in every cross section. The solution is cumbersome, and we do not present it here. Recollect that in the rigid wall case, the pressure is constant in every cross section.

Solutions (119) and (120) are still incomplete as the wavenumber $k=\omega /c$ is still unknown. Consider the mass conservation equation $A_{t}+q_{x}=0$. The flow rate in a cylindrical pipe can be calculated as $q=2\pi \int _{0}^{a}u(r)r\,{\mathrm {d}}\,r$. We write the mass conservation equation as:

121 $$\begin{aligned} A_{t}+2\pi \left( \int _{0}^{a(x)}u(r)r\,{\mathrm {d}}r\right) _{x}=0. \end{aligned}$$

Differentiating the integrand and the upper limit we rewrite it as:

122 $$\begin{aligned} A_{t}+2\pi \int _{0}^{a}u_{x}(r)r\,{\mathrm {d}}r+u(a)A_{x}=0. \end{aligned}$$

In analogy with (115) we can represent the area as:

123 $$\begin{aligned} A = A_0 + \epsilon \delta \tilde{A} \end{aligned}$$

and we rewrite (122) by substituting (112) and (115) into it as

124 $$\begin{aligned}&\epsilon \delta {i}\omega \tilde{A}-\epsilon \delta {i}k\, 2\pi \int _{0}^{a_0 + \epsilon \delta \tilde{a}}[{\hat{u}}+\delta \tilde{u}]r\,{\mathrm {d}}r-\epsilon [{\hat{u}}(a_0 + \epsilon \delta \tilde{a}) \nonumber \\&\quad +\delta \tilde{u}(a_0+ \epsilon \delta \tilde{a})]\epsilon \delta {i}k\tilde{A}=0 . \end{aligned}$$

Retaining the leading terms, we simplify the equation down to

125 $$\begin{aligned} {i}\omega \tilde{A}-{i}k\, 2\pi \int _{0}^{a_0}{\hat{u}}r\,{\mathrm {d}}r=0 . \end{aligned}$$

Intergrading this equation and substituting $\varDelta A = \tilde{A}$ we obtain

126 $$\begin{aligned} \frac{\varDelta A}{A_{0}}=\frac{\varDelta {\hat{p}}}{\rho c^{2}} \left[ 1-\frac{2J_{1}(\varpi )}{\varpi J_{0}(\varpi )}\right] , \qquad \varpi = \varkappa a_{0}. \end{aligned}$$

Parameter $|\varpi |=|\varkappa | a_0$ is called the Womersley number for a given frequency $\omega$. From (24) it follows that in the linear approximation

$$\begin{aligned} \frac{\varDelta A}{A_{0}} = \frac{\varDelta p}{\rho c_0^2}. \end{aligned}$$

then

127 $$\begin{aligned} \frac{\varDelta {\hat{p}}}{\rho c^{2}} \left[ 1-\frac{2J_{1}(\varpi )}{\varpi J_{0}(\varpi )}\right] = \frac{\varDelta p}{\rho c_0^2}. \end{aligned}$$

From Eq. (127) we can find the wave speed corrected by viscous effects as

128 $$\begin{aligned} c^{2} = {c_0^2}{\left[ 1-\frac{2J_{1}(\varpi )}{\varpi J_{0}(\varpi )}\right] } \end{aligned}$$

and the wavenumber

129 $$\begin{aligned} k^{2}=\frac{\omega ^{2}/c_{0}^{2}}{\displaystyle 1-\frac{2J_{1}(\varpi )}{\varpi J_{0}(\varpi )}} . \end{aligned}$$

Using the Womersley solution, we can calculate $\gamma$ for a harmonically oscillating flow with given frequency $\omega$ in cylindrical rigid and flexible pipes as (Fig. 13)

130 $$\begin{aligned} \gamma (\omega )= & \frac{au_r(a)}{u(0)} =\frac{\varpi ^2 J_{1}(\varpi )}{\varpi J_{0}(\varpi )-2J_{1}(\varpi )}\nonumber \\= & 4-\frac{\varpi ^2}{6} +O\left( |\varpi |^4\right) \end{aligned}$$

The fastest way to compute the $\gamma$ parameter is to use Padè approximation. The following approximation gives sufficient accuracy

131 $$\gamma \left( \varpi \right) \approx \left\{ {\begin{array}{*{20}l} {4{\mkern 1mu} \frac{{1 + \frac{1}{{12}}i|\varpi |^{2} - \frac{1}{{960}}|\varpi |^{4} }}{{1 + \frac{1}{{24}}i|\varpi |^{2} - \frac{1}{{5760}}|\varpi |^{4} }}} \hfill & {{\text{if}}\,\left| \varpi \right| < 7.6} \hfill \\ {\frac{8}{5} + |\varpi |\sqrt i } \hfill & {{\text{if}}\,|\varpi | \ge 7.6} \hfill \\ \end{array} } \right.$$

The maximal error is 2.5% at $|\varpi |=7.6$.

## Appendix 2: Algorithm

1. Input the network, blood parameters, outlet parameters, inlet waveform, monitoring site positions: $\{s_m,x_m\}$;
2. Perform FFT for the inlet waveform, and set the highest accounted harmonic *N*.
3. Build the system of equations for the zeroth harmonic with respect to $\{{\hat{P}}_{0s},{\hat{Q}}_{0s}\}$ and solve it.
4. For every nonzero frequency, calculate transmission matrices ${\varvec{T}}_{ns}(x)$ for every vessel and every accounted frequency using (54)–(55) and store them.
5. For every nonzero frequency, build matrix of liner equations with respect to $\{{\hat{P}}_{ns},{\hat{Q}}_{ns}\}$ and a column of the right-hand sides using equations in Sect. 5, and solve the system.
6. Compute the Fourier image $P_{s_mn}(x_m)$ and performing the inverse FFT (75) compute the waveform in the monitoring sites.
7. If accuracy of the linearized problem is enough, terminate the process.
8. Performing inverse FFT (34) compute $p^{(1)}(t,x),q^{(1)}(t,x)$ and also $p_t^{(1)}(t,x),p_x^{(1)}(t,x),q_x^{(1)}(t,x)$ using (77)–(79) in every node.
9. Compute ${\varvec{n}}^{(2)}(t,x)$ in every node using (76).
10. Compute nonlinear corrections $P^{(2)}_{ns}(L_s),Q^{(2)}_{ns}(L_s)$ using (82)–(83) and also (84).
11. Applying (89) compute $U_s^2(0),U_s^2(L_s)$ and then compute ${\mathcal {N}}_{nd}$ using (88).
12. Build the system of linear equations (90) and solve it.
13. Repeat Step 6 and terminate.
